# RNAi-Mediated Down-Regulation of Dicer-Like 2 and 4 Changes the Response of ‘Moneymaker’ Tomato to Potato Spindle Tuber Viroid Infection from Tolerance to Lethal Systemic Necrosis, Accompanied by Up-Regulation of miR398, 398a-3p and Production of Excessive Amount of Reactive Oxygen Species

**DOI:** 10.3390/v11040344

**Published:** 2019-04-13

**Authors:** Takahiro Suzuki, Sho Ikeda, Atsushi Kasai, Akito Taneda, Misato Fujibayashi, Kohei Sugawara, Maki Okuta, Hayato Maeda, Teruo Sano

**Affiliations:** 1 Faculty of Agriculture and Life Science, Hirosaki University, Bunkyo-cho 3, Hirosaki 036-8561, Japan; takahiro.suzuki.ppvd@gmail.com (T.S.); sho.i.hrdi@gmail.com (S.I.); kasaia@hirosaki-u.ac.jp (A.K.); twin.40g.r.grey@gmail.com (M.F.); kohei@t-i-z-z.com (K.S.); okutako3133@yahoo.co.jp (M.O.); hayatosp@hirosaki-u.ac.jp (H.M.); 2 Union Graduate School of Agricultural Sciences, Iwate University, 3-18-8 Ueda, Morioka, Iwate 020-8550, Japan; 3 Graduate School of Science and Technology, Hirosaki University, Bunkyo-cho 3, Hirosaki 036-8561, Japan; taneda@eit.hirosaki-u.ac.jp

**Keywords:** viroid, pathogenicity, RNA silencing, Dicer-like proteins, small interfering RNA, miR398, miR398a-3p, superoxide dismutase, reactive oxygen species, systemic necrosis

## Abstract

To examine the role of RNA silencing in plant defenses against viroids, a Dicer-like 2 and 4 (DCL2&4)–double knockdown transgenic tomato plant line, 72E, was created. The expression of endogenous *SlDCL2*s and *SlDCL4* in line 72E decreased to about a half that of the empty cassette line, EC. When challenged with potato spindle tuber viroid (PSTVd), line 72E showed significantly higher levels of PSTVd accumulation early in the course of the infection and lethal systemic necrosis late in the infection. The size distribution of PSTVd-derived small RNAs was significantly different with the number of RNAs of 21 and 22 nucleotides (nt) in line 72E, at approximately 66.7% and 5% of those in line EC, respectively. Conversely, the numbers of 24 nt species increased by 1100%. Furthermore, expression of the stress-responsive microRNA species miR398 and miR398a-3p increased 770% and 868% in the PSTVd-infected line 72E compared with the PSTVd-infected EC. At the same time, the expression of cytosolic and chloroplast-localized Cu/Zn-superoxide dismutase 1 and 2 (*SOD1* and *SOD2*) and the copper chaperon for SOD (*CCS1*) mRNAs, potential targets of miR398 or 398a-3p, decreased significantly in the PSTVd-infected line 72E leaves, showing necrosis. In concert with miR398 and 398a-3p, SODs control the detoxification of reactive oxygen species (ROS) generated in cells. Since high levels of ROS production were observed in PSTVd-infected line 72E plants, it is likely that the lack of full dicer-likes (DCL) activity in these plants made them unable to control excessive ROS production after PSTVd infection, as disruption in the ability of miR398 and miR398a-3p to regulate SODs resulted in the development of lethal systemic necrosis.

## 1. Introduction

Viroids are the smallest known pathogens of higher plants [1]. They consist solely of a highly-structured, covalently closed, circular RNA molecule that ranges between 246 and 434 nucleotides (nt) in length. The more than 30 species reported so far are classified into one of two genera, *Pospiviroidae* or *Avsunviroidae,* depending on the characteristics of their nucleotide sequence, mode of replication, subcellular localization, host specificity, and specific disease symptoms [2]. Viroids replicate autonomously in the nucleus or chloroplasts of invaded host cells, depending completely on the host’s transcription machinery. Some cause mild to severe disease in sensitive hosts, while other infections are asymptomatic. In light of the non-coding nature of the viroid genome, all the factors necessary to replicate in invaded host cells (i.e., to recruit transcription machinery such as RNA polymerase II and transcription factors, to move from cell to cell, and to spread systemically, and even those necessary to cause disease symptoms) must be embedded in the highly base-paired stem-loop structure [3]. However, many of these properties are yet to be studied and described.

Potato spindle tuber viroid (PSTVd) species of the genus *Pospiviroid* was first identified in North America as the causal agent of spindle tuber disease in potatoes (*Solanum tuberosum*) [1]. Early studies show that PSTVd has a relatively wide experimental host range [4], and, in the early 21st century, natural infections of PSTVd in hosts other than potato were first reported in tomato and ornamentals of the families *Solanaceae* and *Asteraceae* [5,6,7]. Other pospiviroids in addition to PSTVd have begun to expand into new geographical areas through global distribution of contaminated seeds and vegetatively-propagated planting materials, thereby causing serious concerns in the seed industry, as well as plant quarantine, worldwide [8].

The tomato (*Solanum lycopersicum*) is the most sensitive host to PSTVd, but the severity of symptoms varies depending on the host cultivar, as well as the PSTVd variant. For example, in response to a severe isolate of PSTVd, symptomatic cultivar ‘Rutgers’ shows severe leaf curling and stunting, but a tolerant cultivar ‘Moneymaker’ shows little or no stunting, although both accumulate a systemic abundance of PSTVd [9,10]. Such variation in symptoms is believed to be determined by the difference in the genetic makeup of host cultivars in response to the infection of PSTVd variants with distinct molecular characteristics [11].

In response to pathogen attacks, plants induce two layers of defense responses called pathogen-associated molecular patterns (PAMPs)-triggered immunity (PTI) and effector-triggered immunity that involve the activation of various plant defense responses, including programmed cell death [12]. RNA-silencing plays an additional role, functioning to protect many eukaryotic genomes from invading viruses, foreign transgenes, and transposable elements [13,14,15]. For members of the family *Pospiviroidae* that replicate inside the nuclei of host cells, double-stranded or highly-structured regions of RNA molecules synthesized during their replication are recognized by RNA silencing, thereby triggering an attack by dicer-like proteins (DCLs), part of the front line of defense that cleaves the replicated RNA into viroid-derived small RNAs (vd-sRNAs) consisting of 21 to 24 nt [9,16,17,18,19,20,21]. Viroids in the family *Avsunviroidae* replicate in chloroplasts and are also targeted by DCLs and cleaved into small pieces (around 21 to 22 nt) [22,23,24]. Vd-sRNAs are then loaded onto Argonaute (AGO) proteins to form RNA-induced silencing complexes (RISCs), which target native viroid molecules by the guidance of vd-sRNAs and digest them into vd-sRNAs again by slicer activity of AGO proteins [25]. In the meantime, the digested viroid RNA called “aberrant RNA” is speculated to be converted into double-stranded RNA by the action of RNA-dependent RNA polymerase 6 (RDR6) and, again, processed into small pieces by DCLs for the amplification of the silencing signal [26,27]. Finally, the action of RNA-silencing mechanism leads to the accumulation of a large quantity of vd-sRNA in viroid-infected host plants, which is thought to play an important role in various aspects of viroid-host interaction, including defense [28,29,30,31,32], molecular evolution, and even symptom expression [33,34,35,36,37,38,39,40,41,42].

DCLs play a central role in the RNAi pathway and are key components in the biogenesis of small RNAs (sRNAs), called short interfering RNA (siRNA) and micro RNA (miRNA) [43]. Four DCLs have been described in plants: *viz*., DCL1, DCL2, DCL3, and DCL4 [44]. In *Arabidopsis thaliana*, DCL1 is involved in miRNA biogenesis and produces mature miRNAs 21 nt in length [45,46,47].

DCL2 generates stress-related 22 nt natural-antisense-transcript (nat)-siRNAs and 22 nt siRNAs of exogenous origin [48,49] and are reported to process ~22 nt siRNAs from ta-siRNA precursors in the absence of DCL4 [50]. DCL2 (in combination with DCL4) is also involved in the production of secondary siRNAs that trigger a phenomenon known as “transitivity” [51] and play a role in the antiviral defense [48,52,53]. DCL3 produces 24 nt-long, DNA repeat–associated siRNAs that guide heterochromatin formation [48]. DCL4 generates 21 nt-long siRNAs that mediate post-transcriptional silencing of some endogenous genes (e.g., trans-acting (ta)–siRNAs) [50,54] and of transgenes mediating RNA interference [55]. DCL4 is also responsible for processing specific miRNAs in *Arabidopsis thaliana* [56,57] and has a role in transcriptional termination [58,59] and antiviral defense mechanisms [52,60,61]. Among the various roles of DCL proteins, the action to counteract viruses and viroids is particularly interesting, because they are known to work cooperatively, but hierarchically, to combat pathogens in invaded cells as the front line of defense. In particular, DCL2 and DCL4 play important roles in the defense against viruses [60,62]. In addition, DCL1 has the potential to produce 21 nt viral siRNA in the absence of DCL2, DCL3, and DCL4 [52,60].

Published data on the detection of vd-sRNAs in viroid-infected plants suggest that the highly base-paired stem-loop structure of viroids can serve as a substrate for multiple DCLs [9,16,17,18,19,22]. Direct evidence for such cleavage was first obtained from experiments demonstrating that PSTVd RNA was cleaved into small pieces of approximately 21 nt in length when incubated with *Arabidopsis spp.* cell extracts containing DCL activities [18]. A more detailed analysis using a series of *Nicotiana benthamiana DCL*-knockdown lines revealed that PSTVd levels dropped when either (i) *DCL4* expression alone was suppressed, or (ii) *DCL1*, *DCL2*, or *DCL3* were knocked down together with *DCL4*. These observations led to a new hypothesis, that the combined activity of DCL2 and DCL3 is crucial in the defense against PSTVd [20,21]. In this scenario, DCL4 is proposed to play the key role in processing PSTVd, and its activity may obscure or suppress the effects of DCL2/DCL3 on viroid infectivity, suggesting that hierarchical interactions among DCLs are also important in the defense against viroids.

To analyzes PSTVd pathogenicity in tomato plants from the standpoint of RNA silencing, we introduced an inverted-repeat (IR) chimera gene construct, consisting of parts of the tomato *SlDCL2a* and *SlDCL2b genes*, which are highly conserved among all four *SlDCL2*s, and *SlDCL4* into the tomato variety ‘Moneymaker’. We then challenged three lines of T3-generation plants by inoculating them with PSTVd to examine the roles of DCL2 and DCL4 in the defense against viroid infections. In one of these lines (named line 72E), endogenous *SlDCL2*s and *SlDCL4* mRNAs were down-regulated by transgene-derived RNA silencing, the response to PSTVd infection was changed from “tolerant” to “highly susceptible,” and infected plants displayed lethal systemic necrosis. Both deep sequencing analysis and RNA gel-blot hybridization revealed that the size distribution of vd-sRNAs had changed dramatically in PSTVd-infected 72E plants (designated PSTVd-72E), indicating that the defensive capability of DCL2s and 4 against viroid infection was largely impaired. Further analysis revealed that PSTVd infection induced the stress-responsive microRNA species miR398 and miR398a-3p, and that their expression levels were unusually high in PSTVd-72E plants showing necrosis. In response to this, the expression level of mRNAs for cytosolic Cu/Zn-superoxide dismutase 1 (i.e., *SlSOD1* and *SlSOD2* in tomatoes), chloroplast-localized Cu/Zn-SOD2 (i.e., *SlSOD3* in tomatoes), and the copper chaperon for SOD (*CCS1*; i.e., *SlSOD4* in tomatoes), potential targets for miRNA398, 398a-3p, and 398, respectively, declined greatly. Since *SOD*s, including *CCS1* in concert with miR398 and miR398a-3p [63], have a function in controlling the detoxification of harmful reactive oxygen species (ROS) in the cells, their regulation is very important if plants are to tolerate oxidative stresses. In this article, we will present data showing that RNAi-mediated down-regulation of *DCL2*s and *DCL4* results in failed initial defenses against viroid infections, triggers excessive production of ROS by the abnormal expression of miR398 and miR398a-3p regulating SODs, and ultimately results in the development of severe systemic necrosis.

## 2. Materials and Methods

### 2.1. Generation of DCL2/4i Transgenic Tomato ‘Moneymaker’ Lines

**Figure 1:**
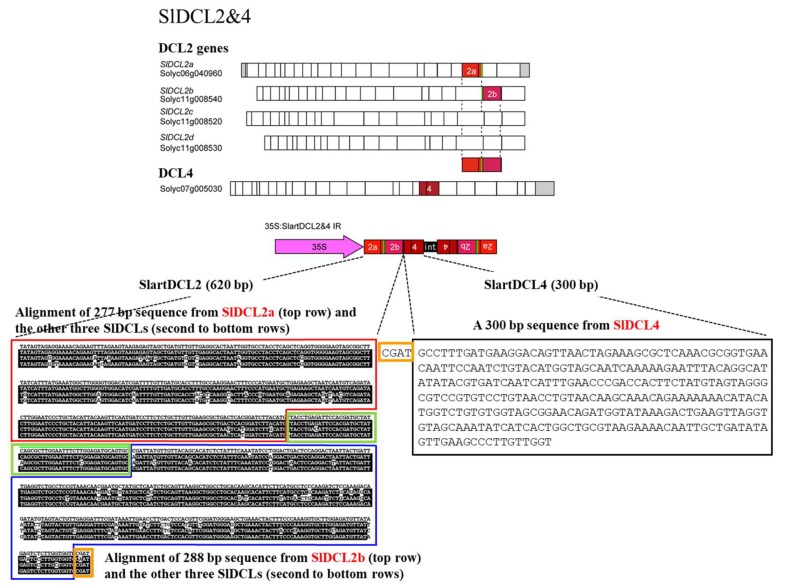
Schematic diagram of the artificial chimera gene (SlDCL2&4). Based on the alignment of four tomato DCL2 genes (*SlDCL2a*, *b*, *c*, *d*) and one DCL4 gene (*SlDCL4*) sequences registered in the database, two regions from *SlDCL2*, i.e., one from *SlDCL2a* (277 bp) and the other from *SlDCL2b* (288 bp), and one region from *SlDCL4* (300 bp), as shown in the top of the figure, were selected. An artificial chimera gene composed of the three sequences from *SlDCL2a* (top row in the red box), *SlDCL2b* (top row in the blue box), and *SlDCL4* (in the black box) was created. The sequences in the second to fourth rows in the red box indicate the corresponding sequences of *SlDCL2b*, *2c*, and *2d*, respectively. The nucleotide with the black background shows the one that is the same as the *SlDCL2a* sequence. Similarly, those in the second–fourth in blue indicate the sequences of *SlDCL2a*, *2c*, and *2d*, respectively. The sequence boxed in green is a region with a common sequence between *SlDCL2a* and *2b*, and that in orange is a sequence common to those of *SlDCL2b* and *4*. A pair of the chimera gene construct were then placed in head-to-head orientation across an intron sequence (‘int’ in the figure) to create an IR sequence. SlartDCL2&4 IR was inserted into the *Sac*II/*Sal*I site of pBluescript II SK (+) plasmid, re-cloned into the *Bgl*II/*Kpn*I site of binary vector pIG121-Hm downstream of the CaMV-35S promoter (35S:SlartDCL2&4 IR), and introduced into *Agrobacterium tumefaciens* strain EHA105 to transform tomato cv. ‘Moneymaker’ by the leaf disc method.

In order to knockdown *DCL2* and *DCL4* expression via RNAi, an IR sequence was constructed as follows, based on the sequence of tomato homologs of *A. thaliana DCL2* (*SlDCL2a*, Solyc06g040960; *SlDCL2b*, Solyc11g008540; *SlDCL2c*, Solyc11g008520; *SlDCL2d*, Solyc11g008530) and *DCL4* (*SlDCL4*, Solyc07g005030) registered in the tomato genome database (https://solgenomics.net/organism/Solanum_lycopersicum/genome). The artificial chimera gene (SlartDCL2&4) was constructed by placing parts of *SlDCL2a* (277 bp), *SlDCL2b* (288 bp), and *SlDCL4* (300 bp) in head-to-head orientation across an intron sequence to create an IR sequence (Figure 1). As shown in the figure, the sequences of *S1DCL2a* and *S1DCL2b* used were selected from regions having high homology with the other *S1DCL2*s sequences to simultaneously knock down all the four *S1DCL2s*. SlartDCL2&4 IR was inserted into the *Sac*II/*Sal*I site of pBluescript II SK (+) plasmid (Agilent Technologies, Tokyo, Japan), re-cloned into the *Bgl*II/*Kpn*I site of binary vector pIG121-Hm [64] downstream of the CaMV-35S promoter (35S:SlartDCL2&4 IR), and introduced into *Agrobacterium tumefaciens* strain EHA105 to transform tomato cv ‘Moneymaker’ by the leaf disc method [28]. Transformants (T0 generation) were selected on media containing kanamycin, transplanted to pots for further cultivation, and self-fertilized to produce the T1 generation. By repeating the kanamycin selection and self-fertilization, three lines (hpDCL2/4i-51-6, -72E, and -82a) of the T3-generation were selected. In addition, tomato plants transformed with pIG121-Hm containing an empty cassette were created and used as a negative control (line EC).

### 2.2. Preparation of PSTVd Inoculum and Infection Assay

Plasmid DNA (~2 μg) containing an infectious cDNA clone of PSTVd-Intermediate (pTZ18R-Rz6-PSTV; accession number M16826) was linearized by *Not*I restriction enzyme digestion (Takara Bio, Otsu, Shiga, Japan) and used for in vitro transcription in a 20 µL reaction mixture containing T7 RNA polymerase (Invitrogen, Carlsbad, CA, USA) at 37 °C for 2 h according to manufacturer’s instruction. Inoculum was adjusted to a concentration of 100 ng of the transcript/μL in 50 mM sodium phosphate buffer (pH 7.5), 1 mg/mL bentonite. For mechanical inoculation, an aliquot (10 μL) was placed on the third true leaf of ‘Moneymaker’ seedlings dusted with carborundum (600-mesh) and gently rubbed against the leaf 10-times using a sterile glass-bar. Ten seedlings each from four different hpDCL2/4i Moneymaker lines (i.e., EC, 51-6, 72E and 82a) were used for each infection assay. After inoculation, plants were incubated in a growth chamber controlled at 22 °C (night)–30 °C (day), 16-h day-length supplemented with high-intensity fluorescent light (40 watts × 4).

### 2.3. Preparation of Total Nucleic Acids, Genomic DNA, and Total RNA from Tomato Plants

The accumulation of viroids in the inoculated plants was examined using total nucleic acids extracted by the CTAB method [65]. Two leaf disks (1 cm in diameter, ~0.05 g) were collected from the uppermost expanded leaves of each plant at two, three, and four weeks post inoculation (wpi), and homogenized in 0.5 mL of 2× CTAB buffer using a multi-specimen cell disruption device (Shake Master, BMS Co Ltd., Tokyo, Japan) with two zirconia-balls (*φ* = 5 mm). Total nucleic acid pellets were dissolved in 50–100 µL distilled water.

The presence and the copy number of transgene were examined by detecting the CaMV-35S promoter sequence in genomic DNA preparations extracted from samples of leaf tissue. Total nucleic acids were extracted from homogenate prepared in 2× CTAB buffer (~1 g/5 mL) with an equal volume (*v*/*v*) of phenol-chloroform (1:1), precipitated by ethanol, and resuspended in 100–400 μL distilled water. They were further incubated with RNaseA (DNase-free, Wako NIPPON GENE, Japan) at 37 °C for 45 min (min) to digest RNAs, and ethanol-precipitated after extraction with phenol-chloroform. RNA-free genomic DNA pellets were dissolved in 100 µL distilled water.

Total RNA preparations required for analysis of transgene transcripts, siRNA derived from transgene transcripts, small RNAs for deep sequencing, and specific host gene expression, were extracted using Trizol (Thermo Fisher Scientific, Tokyo, Japan) or TriReagent (Molecular Research Center, Inc., Cincinnati OH, USA) according to the manufacturer’s instruction.

### 2.4. Detection of CaMV-35S Promoter Sequence by PCR

Portions of the CaMV-35S promoter and actin gene sequences were amplified by PCR using 0.1 µg of total nucleic acids as a template and analyzed by 7.5% polyacrylamide gel electrophoresis (PAGE). PCR was performed using One Taq DNA polymerase (New England BioLabs, Japan) according to the manufacturer’s instruction and the appropriate primer set (see Table S1). The resulting PCR products were fractionated in 7.5% PAGE containing 1× TAE buffer.

### 2.5. Southern-blot Hybridization to Analyse Transgene Copy Number in Transformed Plants

Aliquots of genomic DNA (~15 μg) were digested with *Eco*RI or *Bam*HI (Thermo Fisher Scientific K.K., Japan), electrophoresed at 50V (4V/cm) for 8 h in 1.0% agarose gel (1× TAE buffer), transferred to a nylon membrane (Biodyne; Pall Corporation, Port Washington, NY, USA) after NaOH-denaturation followed by HCl-neutralization, and hybridized with a DIG-labeled cRNA probe for CaMV-35S promoter sequence. Hybridization signals were visualized using Chemidoc-XRS imaging system and quantified using the Quantity One (version 4.6.2) software package.

### 2.6. Northern-Blot Hybridization to Analyse Transgene Transcripts, Sirna Derived from Transgene Transcripts, Vd-Srnas, and Mirna

Total RNA preparations (1~10 μg) extracted using Trizol or TriReagent were denatured by heating for 15 min at 68 °C in a solution containing 50% formamide (for transgene transcripts and host gene expression) or 50% urea (for siRNA derived from transgene transcripts, viroid-specific small RNAs, and miRNA), fractionated in 1.2% agarose gels containing 1× MOPS buffer at 50 V for ~30 min (for transgene transcripts and host gene expression) or 12% polyacrylamide (acrylamide:bisacrylamide = 19:1) gels containing 1× TBE-8M urea at 450V for ~60 min (for siRNA derived from transgene transcripts, viroid-specific small RNAs, and miRNA), transferred to a nylon membrane (Biodyne), and hybridized with DIG-labeled cRNA probes for IR-DCL2/4 transcript, PSTVd, and miR398a-3p (5′-UAUGUUCUCAGGUCGCCCCUG-3′).

### 2.7. RT-qPCR Analysis of Endogenous DCLs, SODs, and miR398a-3p Expression Levels

Total RNA was extracted using TRizol reagent (Invitrogen, USA), and treated with TURBO DNA-free (Applied Biosystems, Ambion, USA). In accordance with the manufacturer’s instructions, cDNA was synthesized from 1 µg RNA as a template using Superscript VILO (Invitrogen). qPCR analysis was performed essentially as described in Kasai et al. (2013) [31] using SsoFastEvaGreen Supermix (Bio-Rad) with a Chrome4 real time PCR detector (Bio-Rad). Information used to design the PCR primers for tomato *DCL1*, *DCL2*s, *DCL3*, and *DCL4* genes was obtained from the EMBL database. The PCR primers for tomato cytosolic Cu/Zn-SOD1 (i.e., *SlSOD1* and *SlSOD2*), chloroplast-localized Cu/Zn-SOD2 in (i.e., *SlSOD3*), and copper chaperon for superoxide dismutase CCS1 (i.e., *SlSOD4*) were those reported in Feng et al. [66]. The RT-qPCR results were normalized to an actin gene, because in our preliminary examinations, the gene was most stably expressed in our experimental conditions during the observation period. The primers used for qPCR are described in Table S1. For the analysis of miR398a-3p by RT-qPCR, stem–loop reverse transcription followed by TaqMan PCR analysis [67] were applied using primers and probes listed in Table S1.

### 2.8. sRNA Preparation and Deep Sequencing Analysis of PSTVd-Derived sRNAs

Samples of leaf tissue (a total of ~1 g) were collected from two leaves each (leaves 5 and 6 showing yellowing and necrosis) from three plants each (i.e., a total of six leaf tissues) of line 72E and EC infected with PSTVd at three wpi. Total RNAs extracted by Trizol were quantified by UV spectrophotometry and sent aliquots (ca. 50 μg) to Hokkaido System Science Co., Ltd. (Sapporo, Japan) for small RNA sequence analysis (2Gb scale, paired end) on an Illumina HiSeq (Illumina, San Diego, CA, USA). Samples were quantified and their integrities verified using an Agilent 2100 Bioanalyzer (Agilent Technologies, Santa Clara, CA, USA), and processed using a TruSeq small RNA Library Prep Kit.

Adapter sequences were removed from the ends of the resulting raw short-read data based on the presence of an exact 10 nt match with the termini of the respective adapters, and identical short reads were grouped according to read size (15–45 nt). In this way, adapter-trimmed short read data was converted to a non-redundant and also a redundant “short-read-sequence occurrence” format. The redundant data allowing a maximum of 1 nt mismatch were then mapped to either the genomic or anti-genomic strand of the circular form of PSTVd genome using hssmap, a specially-written C language program to process the data. The non-redundant data, on the other hand, were used for host small RNA analysis including miRNAs after normalization to the number of reads per million reads.

MiRNA analysis was performed manually using the latest miRbase (Release 21). A list of sequences that includes precursor miRNAs together with annotations for mature regions in miRNA was downloaded from miRbase (http://www.mirbase.org/).

### 2.9. ROS Production and Scavenging Activity Assay

The generation of ROS was analyzed by quantitating hydrogen peroxide using a commercial kit Radical catch (Hitachi Ltd., Tokyo, Japan). Briefly, a leaflet (ca. 0.2 g) was homogenized in 1 mL of 0.1 M sodium phosphate buffer (pH 7.0) and centrifuged at 13,000 rpm for 5 min to collect the supernatant. According to the manufacturer’s instructions, an aliquot of the supernatant (10 µL) was mixed with a mixture of 5 mM of cobalt chloride solution (Reagent A; 25 µL) and luminol solution (Reagent B; 25 µL), and reacted for 120 s (s) to measure the amount of luminescence emission in an incubator (AccuFLEX Lumi400; Hitachi Ltd., Tokyo, Japan). Sample luminescence and the control luminescence were obtained by subtracting the measured value of 80 s from that of 120 s. Homogenization buffer was used for control. Statistical analysis was performed using R software.

ROS scavenging activity was analyzed by quantitating hydrogen peroxide scavenging activity using the same kit. Briefly, the supernatant obtained above was further diluted 50-times with the same buffer. According to the manufacturer’s instructions, an aliquot of the diluted supernatant (10 µL) was mixed with a mixture of 5 mM of cobalt chloride solution (Reagent A; 25 µL) and luminol solution (Reagent B; 25 µL), and incubated at 37 °C for 5 min. The mixture was further added by hydrogen peroxide solution (1:1000-diluted Reagent C; 25 µL) and reacted for 120 s to measure the amount of luminescence emission. Sample luminescence and the control luminescence were obtained by subtracting the measured value of 80 s from that of 120 s. Homogenization buffer was used for control. Hydrogen peroxide scavenging activity was calculated using the following equation; i.e., Hydrogen peroxide scavenging activity (%) = {Luminescence (Control) − Luminescence (Sample)}/Luminescence (Control) × 100. Statistical analysis was performed using R software.

## 3. Results

### 3.1. Characterization of DCL2/4-Knockdown Transgenic Tomato Lines

The hairpin RNA produced by transcription of the SlartDCL2&4 IR transgene in tomato cells should activate the RNAi machinery, induce the production of siRNAs complementary to *SlDCL2a*, *SlDCL2b*, and *SlDCL4* transcripts, and suppress endogenous *SlDCL2s* and *SlDCL4* expression. To assess the effects of transgene expression, we first verified the presence of transgene, the transgene copy number, the expression level of transgene transcript, and the accumulation of siRNAs derived from the transgene transcript in the three lines selected for the study.

Presence of the transgene was examined by PCR amplification of CaMV-35S promoter sequence from genomic DNA extracted from transgenic lines. As a result, an amplicon of approximately 900 bp, the size expected from the primer set used, was detected from all the three lines of hpDCL2/4i (Figure S1a).

The transgene copy number was assayed by Southern-blot hybridization using a DIG-labeled cRNA probe for the CaMV-35S promoter. From line 51-6, two bands were detected in an *Eco*RI-digest, and one band in the corresponding *Bam*HI digest, indicating that this line contains two copies. From line 72E, one band was detected in both the *Eco*RI and *Bam*HI digests, indicating that this line contains a single copy. Multiple (5–6) bands were detected in both the *Eco*RI and *Bam*HI digests of line 82a, indicating that this line contains multiple copies of the transgene (Figure S1b).

Transgene transcripts were assayed using Northern hybridization with a DIG-labeled cRNA probe for SlartDCL2&4. A dense positive signal was detected from 1 μg of total RNA in line 72E, indicating the accumulation of high levels of transgene transcript. On the other hand, the signal was virtually invisible in samples from lines 82a and 51-6 as well as in the negative control line EC, even when higher amounts (10 μg) of total RNA were loaded (Figure S1c).

The level of siRNA accumulation derived from the transgene transcript was analyzed by Northern-blot hybridization using the same probe as above. In lines containing the transgene, a positive signal with a size of approximately 20 nt was detected only in line 72E; no signals were visible for lines 51-6, 82a, and EC (Figure S1d).

### 3.2. DCL2/4-Knockdown Transgenic Tomato Line 72E Developed Severe Disease Symptoms upon PSTVd Infection

Line 72E contains a single copy of the transgene and expresses high levels of transgene transcript and their corresponding siRNA. Therefore, changes in the appearance of line 72E plants in response to PSTVd infection were compared to those seen in other lines.

**Figure 2:**
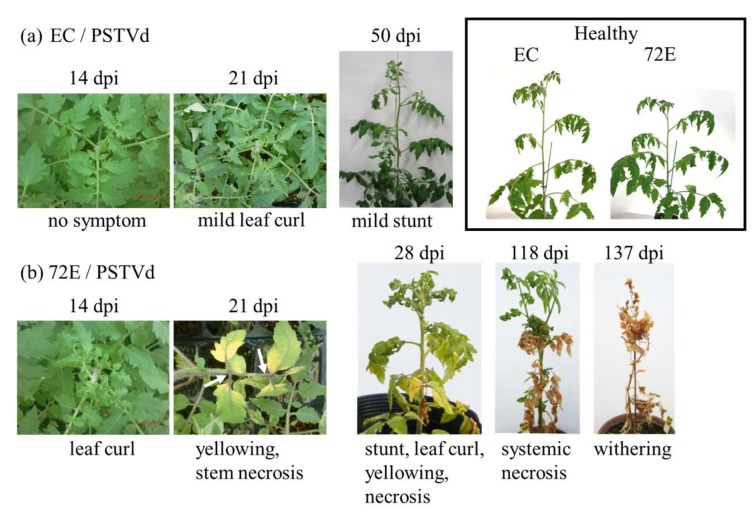
Lethal necrotic symptoms associated with potato spindle tuber viroid (PSTVd) infection in the DCL2/4i-72E ‘Moneymaker’ tomato line. The upper row (**a**) indicates line EC infected with PSTVd. Plants showed mild leaf curling ~21 dpi and mild stunting at 50 dpi. The lower row (**b**) indicates line 72E infected with PSTVd. Infected plants started to show yellowing and stem necrosis around 3–4 weeks after inoculation, then showed severe systemic necrosis around 2–3 months post inoculation, and finally stopped growing and died. Plants in the box are healthy lines 72E and EC (~50 dpi). Three replicates of plants showed similar symptoms. Photos were taken on the days indicated above the pictures.

Ten plants each from lines EC and 51-6 showed very mild leaf curl four weeks after PSTVd inoculation and continued to grow, showing only slight growth retardation compared to the uninoculated healthy controls. In contrast, all ten plants of line 72E started to show curling on the newly expanding apical leaves two weeks post inoculation (wpi); this curling then became more severe, chlorosis appeared on the expanded leaves, and the mid-veins and/or petioles became necrotic around 3 wpi. The symptoms rapidly worsened between 3–4 wpi (Figure 2a,b and Figure S2). Nine plants of line 82a (even though the expression of neither transgene transcript nor siRNA was evident) showed mild leaf curl and chlorosis at 3 wpi (Figure S2), symptoms that were milder than those on line 72E. Continued observation of infected line 72E plants revealed that growth almost stopped around 4 wpi, the severe necrosis first observed in the lower leaves became systemic, and, finally, the plants died three to four months post inoculation (Figure 2b). The infection assays were performed three-times under the same conditions and the results were in agreement with those above.

### 3.3. High Levels of PSTVd Accumulated in Line 72E in the Early Stages and PSTVd Differentially Accumulated in Transgenic Lines

Total RNA extracted from the upper leaves of nine to ten individual tomato plants from each line at 2, 3, and 4 wpi was used to monitor the levels of PSTVd accumulation by Northern-blot hybridization using a DIG-labeled PSTVd-cRNA probe. PSTVd was detected in almost every inoculated plant, even at 2 wpi (10/10 plants infected in line EC, 8/10 in line 51-6, 10/10 in line 72E, and 9/9 in line 82a). One or two weeks later, all plants were infected (Figure S3).

**Figure 3:**
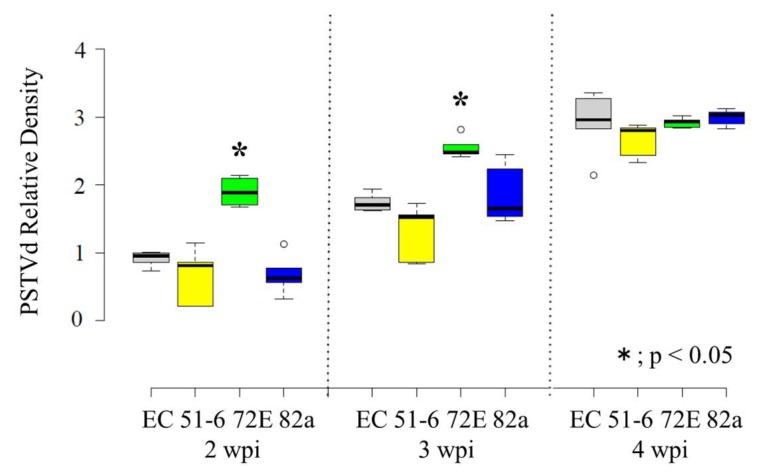
The relative density of PSTVd in three lines of the DCL2/4i Moneymaker tomato and the empty cassette control line at 2, 3, and 4 wpi. Five replicates of PSTVd-positive signals obtained by Northern-blot hybridization were quantified by ChemiDoc XRS, normalized using the gel image stained with ethidium bromide, and the averages were plotted on the graph. The thin vertical lines on the top indicate the error bar. The average value of the empty line at 2 wpi was adjusted as 1.0. The value in line 72E at 2 and 3 wpi with asterisks (*) were statistically significant at 5% level (*p* < 0.05). Boxplots were drawn in BoxplotR [69] using the Tukey whisker extent. Center lines show the medians; box limits indicate the 25thand 75thpercentiles as determined by R software; whiskers extend to 1.5-times the interquartile range from the 25th and 75th percentiles, and outliers are represented by dots. *n* = 3 sample points.

The intensity of the PSTVd-positive signal was visibly higher in line 72E compared to the other lines, especially at 2 and 3 wpi (Figure S3). Therefore, the intensities of each signal were quantified using the Quantity One software (BioRad), normalized by comparison with the signal intensities obtained with ethidium bromide staining, and then averaged per line per week. Finally, relative intensities were calculated by defining the average value of line EC at 2 wpi as 1.0. These calculations confirmed that relative accumulation levels were actually higher in line 72E early in the infection: From high to low at 2 wpi, lines 72E (~2.1), EC (1.0), 82a (~0.75), and 51-6 (~0.7); at 3 wpi, lines 72E (~2.8), 82a (~2.1), EC (~1.9), and 51-6 (~1.45) (Figure 3). Furthermore, although PSTVd concentrations in line 72E were twice as high as those in the other lines at 2 to 3 wpi, progeny levels were approximately the same later in the infection (lines 82a (~3.3), 72 E (~3.2), EC (~3.2), and 51-6 (~3.0) at 4 wpi). Relative accumulation levels were somewhat lower in lines 82a and 51-6 at 2 wpi than that in line EC, but further examination is necessary to see whether this has important meaning.

The complete nucleotide sequences of PSTVd progenies propagated in lines 72E and EC were examined by using cDNA clone-sequencing (10 each of cDNA clones) and by analysis using the deep sequencing data of PSTVd-sRNAs, as described by Suzuki et al. [68], and found that they were virtually identical to the original sequence infected, although some minor singleton mutations, probably raised by replication error or misincorporation during PCR, were detected.

### 3.4. PSTVd Infection Activates DCLs

**Figure 4:**
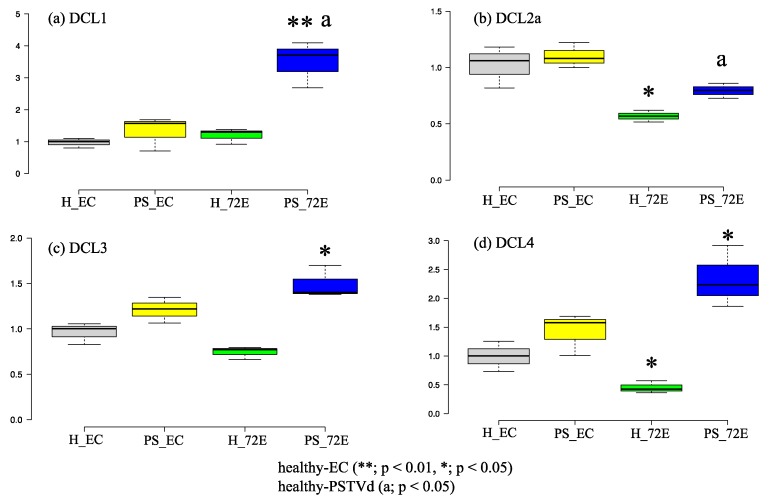
RT-qPCR analysis of endogenous *SlDCL1*, *2a*, *3*, and *4* mRNAs in lines 72E and EC with or without infection of potato spindle tuber viroid (PSTVd) using the leave at 3 wpi. Levels of *SlDCL2a* and *4* mRNAs in line 72E were significantly lower compared to those in the empty cassette before PSTVd infection, suggesting that endogenous *SlDCL2a* and *4* mRNAs were successfully down-regulated in line 72E (**b**,**d**). Levels of mRNAs of *SlDCL1*, *3* and *4* were significantly up-regulated in line 72E by PSTVd infection (**a**,**c**,**d**). Surprisingly, *SlDCL2a* was also up-regulated in line 72E compared to the healthy line, suggesting that the expression of tomato DCL genes are significantly activated by PSTVd infection (**b**). The assay was repeated twice, and each analysis consisted of three biological replicates collected from three plants per treatment. The values with the double asterisk (**; *p* < 0.01) and single asterisk (*; *p* < 0.05) were statistically significant at 1% and 5% levels compared to healthy-EC. The values with “a” (a; *p* < 0.05) were statistically significant at the 5% level compared to PSTVd-EC.

The levels of *SlDCL*s *(1*, *2a*, *3*, and *4*) transcripts before and after PSTVd infection in line 72E were analyzed by RT-qPCR (Figure 4). Before PSTVd infection, the levels of *SlDCL2a* and *SlDCL4* transcripts in healthy line 72E plants were significantly lower (*p* < 0.05) than those in the healthy line EC plants. In contrast, levels of *SlDCL1* transcripts were almost identical, and levels of *SlDCL3* transcripts were slightly (but not significantly) lower in line 72E. This result, as expected, indicated that expression of the *SlDCL2a* and *SlDCL4* genes had been down-regulated in line 72E by RNAi before PSTVd infection. In contrast, transcript levels of *SlDCL1*, *2a*, *3,* and *4* were all significantly up-regulated in line 72E after PSTVd infection. The increase in *SlDCL1* was especially remarkable (*p* < 0.01). This was also true for line EC, indicating that transcription of *SlDCL1–4* was activated by PSTVd infection but that changes were somewhat bigger in line 72E than in line EC.

Furthermore, since the nucleotide sequence of SlDCL2a used for the transgene was very similar to those of SlDCL2b (89%), SlDCL2c (93%), and SlDCL2d (90%) (Figure 1), the expression levels of these SlDCL2s genes were also examined. As a result, the level of mRNA of SlDCL2d was significantly low in line 72E (~60% of that of EC) compared to that in line EC before PSTVd infection, and up-regulated after infection but still ~70% of that of line EC (Figure S4a). On the other hand, levels of mRNAs of SlDCL2b and SlDCL2c were considerably lower either in line EC and 72E before and after PSTVd infection (Figure S4b,c), suggesting that the expression of these genes was originally very low.

### 3.5. Changes in PSTVd-Derived sRNA in PSTVd-72E; PSTVd-sRNA of 21 and 22 nt Species Decreased and 24 nt Species Increased in the Transgenic Line 72E in Response to the Knockdown of DCL2 and DCL4

#### 3.5.1. Northern-Blot Hybridization Analysis

**Figure 5:**
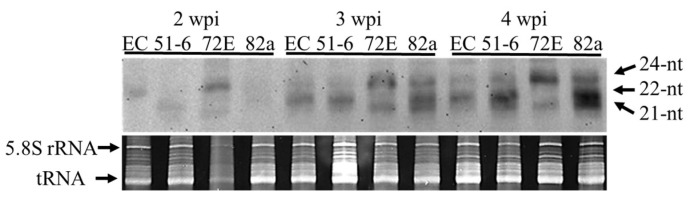
Time course analysis of PSTVd-sRNA accumulating in PSTVd-infected DCL2/4i-transgenic tomato lines. Total RNAs (~10 µg) extracted from three transgenic lines inoculated with PSTVd and line EC at 2, 3, and 4 wpi were separated in 8M-urea 12% PAGE, transferred to nylon membrane, and analyzed by RNA gel-blot hybridization analysis using a DIG-PSTVd-cRNA probe. The upper panel shows the hybridization signal and the lower panel shows the loading control stained with ethidium bromide. All the lines except for 72E showed a dense hybridization signal at the position 22 nt and faint signals at 21 and 24 nt. In contrast, line 72E showed a dense signal at 24 nt and a faint signal at 21 nt. The signal at 22 nt was invisible.

In order to analyze the accumulation of PSTVd-derived sRNA (PSTVd-sRNA), groups of RNA extracts from nine to ten plants collected at weekly intervals were combined, aliquots (~10 µg) were fractionated by electrophoresis in an 8M-urea 12% PAGE, transferred to nylon membrane, and hybridized with a DIG-labeled PSTVd-cRNA probe. Results revealed detectable levels of PSTVd-sRNA in lines EC and 72E even at 2 wpi; their size distributions were quite different, however. In the negative control line EC, the predominant species was 22 nt. In line 72E the major peak was two nucleotides longer (24-nt). This difference could be seen more clearly in the 3- and 4-week samples. Here, lines EC, 51-6, and 82a all accumulated abundant 22 and/or 21 nt species and a small amount of 24 nt species. Line 72E, in contrast, accumulated higher amounts of 24 nt species and trace levels of 21 nt species; the 22 nt species was virtually invisible in this sample (Figure 5). Note also that accumulation of PSTVd-sRNA increased in parallel with an increase in PSTVd genome RNA accumulation over 2–4 wpi.

The difference in the size distribution of PSTVd-sRNAs observed in line 72E is consistent with the suppression of *SlDCL2*s and *SlDCL4* expression by RNAi in the line. That is, the decrease in 21 nt species can be explained by down-regulation of *SlDCL4* which is responsible for producing 21 nt sRNAs. Similarly, the decrease in 22 nt species is consistent with down-regulation of *SlDCL2*s responsible for the production of 22 nt species. It should be noted here that the observed decrease in 21 nt species was not as pronounced as that in 22 nt species.

#### 3.5.2. Deep Sequencing Analysis

It is generally accepted that vd-sRNA processed by multiple DCLs activities after induction of RNA silencing may play a major role in viroid pathogenesis or symptom expression. Thus, we carried out deep sequencing analysis of sRNAs prepared from PSTVd-72E and compared the changes in PSTVd-sRNA, host miRNAs, and the other sRNAs with those observed in PSTVd-EC. The sRNA data sets obtained by Illumina Hiseq small RNA analysis contained a total 23,604,108 reads ranging in size from 15–45 nt in PSTVd-72E sampled at 3 wpi and 23,864,986 reads in the comparable sample from PSTVd-EC.

Allowing a maximum of 1 nt mismatch, PSTVd-sRNA sequences accounted for 678,643 reads in PSTVd-72E and 554,261 reads in PSTVd-EC samples. PSTVd-sRNAs in PSTVd-72E comprised 383,947 reads (69%) from plus-strand and 170,091 reads (31%) from the minus-strand., i.e., sRNAs derived from the plus-strand were slightly more than twice as abundant as those from the minus-strand (Figure S5). This ratio was very similar in line EC; i.e., 318,585 reads (63%) to 188,092 reads (37%).

**Figure 6:**
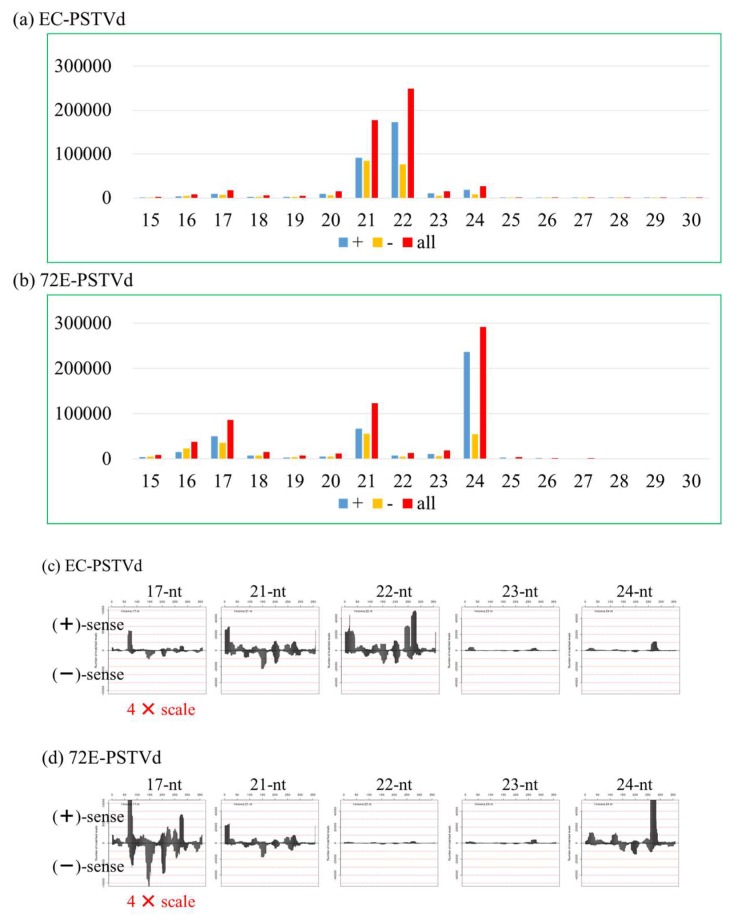
Size distribution and positive (+)/negative (–) ratio of PSTVd-sRNAs of the size 15–30 nt accumulated in lines (**a**) EC and (**b**) 72E. Panels (**c**) and (**d**) compare the PSTVd-sRNA hotspot patterns for the 17 nt, 21 nt, 22 nt, 23 nt, and 24 nt species in lines EC (c) and 72E (d).

In contrast, and as expected from the data obtained by RNA gel-blot hybridization, the size distributions of PSTVd-sRNAs in these two lines were quite different. In line EC, the most abundant size class was 22 nt (50%), followed by 21 nt (36%), 24 nt (5%), and 23 nt (5%), a result which corresponded to our previous data obtained from PSTVd-infected ‘Rutgers’ tomato [9,70]. In PSTVd-72E, however, the most abundant size class was 24 nt (53%), followed by 21 nt (23%), 17 nt (18%), 23 nt (4%), and 22 nt (3%) (Figure 6a,b). These results were quite consistent with the data obtained by RNA-gel blot assay described above. That is, by knocking down DCL2s and DCL4 expression using an RNAi strategy, the number of PSTVd-sRNA reads containing 22 nt in PSTVd-72E dropped sharply to levels about one-twentieth of those seen in line EC plants (i.e., from 253,425 reads in PSTVd-EC to 14,257 in PSTVd-72E), but the number in the 24 nt class increased approximately 10-fold (i.e., from 27,255 reads in PSTVd-EC to 295,542 reads in PSTVd-72E). The number in the 21 nt class also decreased in PSTVd-72E, but this decline was much smaller than in the 22 nt class, even though expression of DCL4, as well as DCL2s, was knocked down, suggesting that DCL1, in addition to DCL4, plays an important role in the production of 21 nt PSTVd-sRNA (see Discussion session).

Interestingly, molecules in the 17 nt class accounted for 18% of the total PSTVd-sRNA reads and were ranked the third most abundant class in PSTVd-72E. At present, it is not clear why the 17 nt class increased so extensively in line 72E, and the possible function of the 17 nt class has yet to be determined. It should be noted here that the majority of these molecules originate from the upper portion of the pathogenicity region; furthermore, the most abundant member of this class, which accounted for 17,606 reads (~19.2%) corresponds to nucleotides 63–79 in the PSTVd genome (5′-AGGCGGCUCGGAGGAGC-3′).

Although the size distributions of PSTVd-sRNA were quite different for PSTVd-72E and PSTVd-EC, the hotspot patterns were virtually identical. This was most evident for the 21 nt species in which the total numbers of reads for the two lines were not significantly different (Figure 6c,d). Interestingly, this was also the case for the 22 nt and 24 nt species where the total numbers of PSTVd-sRNA reads were significantly different; i.e., it was clear that the hotspot patterns of PSTVd-sRNAs in PSTVd-72E and PSTVd-EC were virtually identical when the scales of vertical axis were normalized.

Note also that the hotspot patterns seen in this analysis were very similar to those from PSTVd-infected ‘Rutgers’ tomato presented in our earlier report [11], indicating a high degree of reproducibility among multiple deep sequencing analyses (Figure S6). The fact that the hotspot pattern of PSTVd-sRNA remained constant even when the size distribution changed substantially suggested that even a significant change in the hierarchical order of DCL function caused by RNAi-mediated down-regulation of DCL2s and DCL4 expression was not sufficient to change the potential recognition sites and cleavage activities of the individual DCLs.

### 3.6. Changes in Host sRNAs and miRNA Expression Levels in PSTVd-72E and -EC; PSTVd Infection Up-Regulated miR398 and miR398a-3p

In PSTVd-72E, expression levels of all four *DCL*s were altered, and, as a result, the size distribution of PSTVd-sRNA changed extensively. Also, because the overall response to PSTVd infection in line 72E changed from tolerance to high sensitivity, relative levels of miRNAs and other host-derived sRNA species in this line were compared with those in PSTVd-EC plants. As described above, these comparisons were made using 21 nt species in which the total number of reads did not change so much between lines 72E and EC. After the normalization to the ratio per million reads, comparisons of all sRNAs present at levels more than 50 reads per million revealed that 12 species of miRNAs were up- or down-regulated by more than three-times in PSTVd-72E. The data presented in Table 1 reveal that nine species of miRNAs were up-regulated, whereas three species were down-regulated. In particular, miR398 and miR398a-3p showed unusually high expression levels in PSTVd-72E, where the number of reads per million increased from 311 to 2080 and from 1922 to 16,676, respectively. As described below, these miRNAs are known to target the mRNA of superoxide dismutase which removes harmful ROS from the cell.

As shown in Table 1, several sRNAs of host origin, other than miRNA species, were also detected. Some of these greatly fluctuated in the number of reads in PSTVd-72E, but the others did not fluctuate (Table 1). The top five (i.e., miR166b, 25S rRNA (LOC108175346), miR159a precursor RNA, miR166c, and miR396b) were almost the same in order; i.e., a difference was only seen in the order of the 4th and 5th places. Interestingly, 12 of the top 50 sRNAs in PSTVd-72E were those derived from *SlDCL2*s (9 species with 7596–1775 reads/million) and *SlDCL4* (3 species with 6845–2019 reads/million); however, they were approximately zero in PSTVd-EC. Therefore, it was confirmed that RNAi-mediated digestion of transgene-derived and/or endogenous transcripts of *SlDCL2a* and *SlDCL4* genes actually took place in line 72E plants. Of particular interest was that not only siRNAs of *SlDCL2a* but also a large number of those that could have been derived from *SlDCL2c* and *SlDCL2d* were also detected in PSTVd-72E (Table 1). That is, as described above, since a highly similar-region of *SlDCL2*s was used for the construction of a transgene, it was suggested that inhibitory action was exerted not only on *SlDCL2a* but also on the other mRNAs of *SlDCL2*s; i.e., *SlDCL2c* and *SlDCL2d* at least. As described, cross-inhibition of expression of the other *SlDCL*s by the *SlDCL2a* and *2b* transgene was confirmed in *SlDCL2d* by RT-qPCR analysis.

In addition, it should be noted here that five species of 21 nt sRNAs derived from 25S rRNA decreased in number extensively from 8,109–1,739 to 130–37, Sly-PHAS16 precursor siRNA decreased from 3,158 to 14, and Sly-PHAS04 precursor siRNA decreased from 1545 to 1.

### 3.7. Northern-Blot Hybridization of miR398a-3p in Healthy and PSTVd-Infected Lines 72E and EC; PSTVd Infection Up-Regulated miR398a-3p

**Figure 7:**
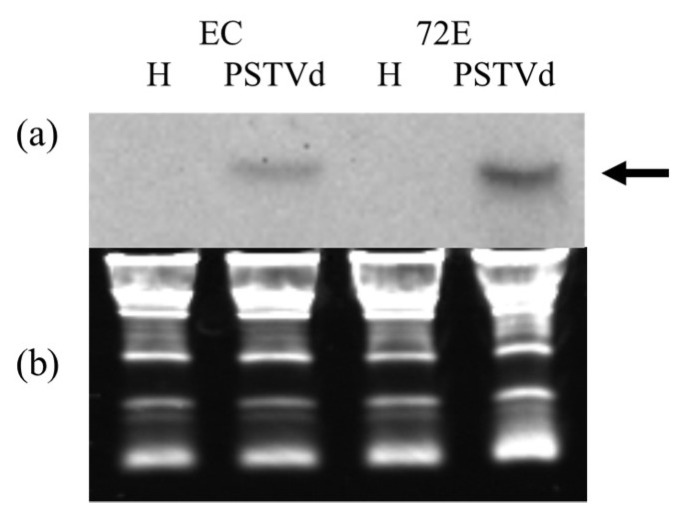
Northern-blot hybridization of miR398a-3p. Aliquots (10 μg) of total RNA, isolated at 3 wpi, were fractionated by electrophoresis in 8M-urea 12% PAGE, transferred to nylon membrane, and hybridized with a DIG-labeled cRNA probe for miR398a-3p. (**a**) Band signals were visualized with ChemiDoc XAR (BioRad) and quantified with Quantity One software (Bio-Rad). MiR398a-3p was detectable only after PSTVd infection (arrow), and signal intensities were approximately five-times higher in line 72E as compared to a control line EC. (**b**) The loading control stained with ethidium bromide. This analysis was repeated twice.

Since the greatest increase was seen in miR398a-3p by deep sequencing analysis, the expression levels of miR398a-3p after PSTVd infection were also examined by Northern-blot hybridization. Aliquots (10 µg) of total RNA, extracted from healthy and PSTVd-infected lines 72E and EC were fractionated by 8M-urea 12% PAGE, transferred to a nylon membrane, and hybridized with a DIG-labeled cRNA probe for miR398a-3p. As shown in Figure 6, miR398a-3p was not detectable in healthy plants from either line, indicating that expression levels of miR398a-3p were very low. In contrast, in PSTVd-infected plants, miR398a-3p reached detectable levels at 3 wpi (Figure 7a, arrow), indicating that PSTVd infection stimulates expression of miR398a-3p. In agreement with the deep sequencing data, the level of miR398a-3p in PSTVd-72E was approximately five times higher than that in the comparable PSTVd-EC plants, reconfirming that RNAi-mediated down-regulation of DCL2s and DCL4 results in enhanced expression. This analysis was repeated twice and yielded similar results.

### 3.8. Expression of Tomato SODs in Healthy and PSTVd-Infected Lines 72E and EC

In line 72E, infection with PSTVd caused severe systemic necrosis and induced unusually high levels of expression of miR398 and miR398a-3p. MiR398 is a miRNA that negatively regulates SOD, which normally scavenges harmful ROS. When *Arabidopsis thaliana* is exposed to oxidative stress arising from biotic or abiotic factors, miR398 expression is down-regulated at the level of transcription, and as a result, the post-transcriptional accumulation of Cu/Zn-SOD1 and Cu/Zn-SOD2 mRNAs is up-regulated, leading to the scavenging of ROS. This is an important regulatory function that enables plants to tolerate oxidative stresses [71]. Therefore, in order to investigate what is happening between these stress-responsive miRNAs and tomato SOD gene expression in the PSTVd-infected line 72E, the target sequences of tomato miR398 and miR398a-3p detected in this experiment were searched for in the tomato unigene database using psRNATarget software (URL; http://plantgrn.noble.org/psRNATarget/). As a result, the target sequences of miR398 were found in the gene sequences of cytosolic Cu/Zn-SOD1 (accession number SGN-U581590) and the copper chaperon for SOD, namely CCS1 (accession number SGN-U577152) (Figure S7). In addition, the target sequence of miR398a-3p was found in the gene sequence of chloroplast-localized Cu/Zn-SOD2 (Figure S7). Recently, Feng et al. [66] identified nine SOD genes (*SlSOD1* to *SlSOD9*) in tomato plants, and reported that *SlSOD1*, *SlSOD2*, *SlSOD3*, and *SlSOD4* all have Cu/Zn-SOD motifs, and that *SlSOD1* and *SlSOD2* are cytosolic whereas *SlSOD3* is chloroplast-localized. It is therefore considered that *SlSOD1* and *SlSOD2* belong to what are referred to as cytosolic Cu/Zn-SOD1s, and *SlSOD3* belongs to chloroplast-localized Cu/Zn-SOD2. Using a BLAST search analysis, the target sequence of miR398 was confirmed in the gene sequence of *SlSOD1* and *SlSOD2*, and the target sequence of miR398a-3p was confirmed in the gene sequence of *SlSOD3*. In addition, *SlSOD4*, which is reported to have Cu/Zn and heavy-metal binding motifs [66] has a nucleotide sequence similar to the *CCS1* sequence reported previously (accession number NM_001347085, AK319564) and has the target sequence of miR398. Based on these findings, correlations among expression levels of miR398a-3p, *SlSOD1*, *SlSOD2*, *SlSOD3*, and *SlSOD4* were analyzed using RT-qPCR. MiR398a-3p was selected for analysis because it was approximately eight-times more abundant than miR398 (Table 1).

**Figure 8:**
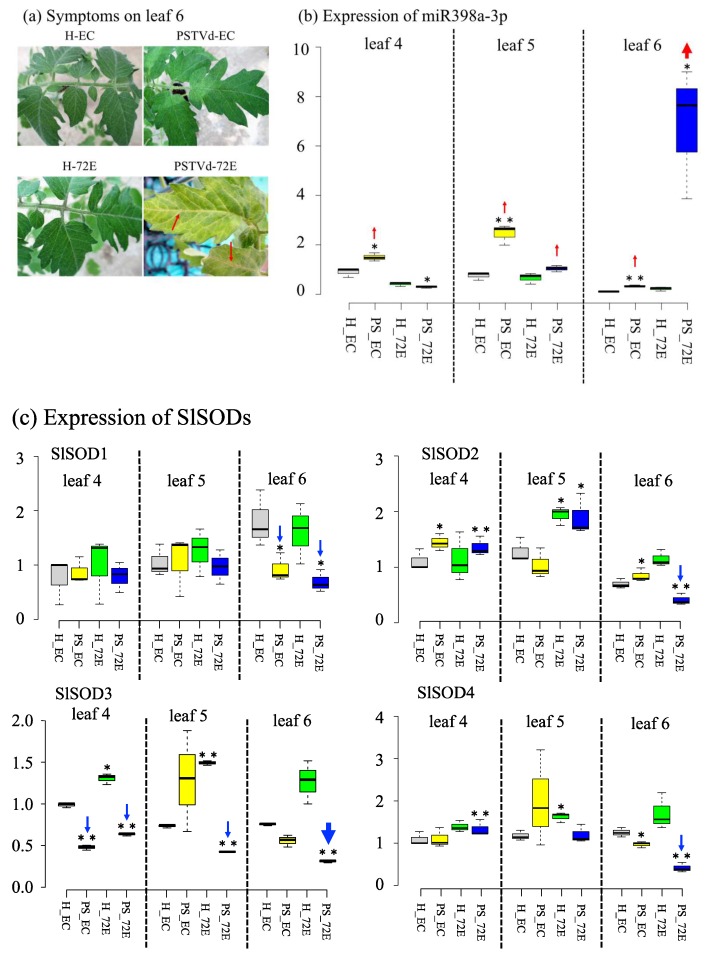
Analysis of miR398a-3p and tomato SODs expression levels in healthy leaves and PSTVd-infected leaves with or without showing necrosis. Panel (**a**): Three plants each of tomato lines 72E and EC were infected with PSTVd. At 4 wpi, only PSTVd-72E (PS_72E) started to show yellowing accompanied with vein necrosis (arrows) on leaves 5 and 6 (numbers from the bottom). Panel (**b**): RT-qPCR analysis of miR398a-3p expression revealed that levels of miR398a-3p were unusually high in leaf 6 of PS_72E, which showed necrosis (**thick red arrow**). Relatively high levels of expression were also found in leaves 4 and 5 in PSTVd-EC (PS_EC) and in leaf 5 in PS_72E (**thin red arrow**), but the levels were approximately four to five times lower than those in leaf 6 of PS_72E. Samples were collected at 4 wpi. The assay was repeated twice, and each analysis consisted of three biological replicates collected from three plants per treatment. Panel (**c**): RT-qPCR analysis of the expression of four tomatoes Cu/Zn-SODs (i.e., *SlSOD1*, *SlSOD2*, *SlSOD3*, and *SlSOD4*) in the same samples revealed that, in response to the highest level of miR398a-3p, *SlSOD3* expression decreased most significantly in leaf 6 of PS_72E (**thick blue arrow**) compared with the healthy line EC (H_EC). *SlSOD3* expression levels were also significantly decreased in leaves 4 and 5 of PS_72E, and in leaf 4 of PS_EC. In addition, *SlSOD1*, *SlSOD2*, and *SlSOD4* expression levels were significantly lower in leaf 6 of PS_72E (**thin blue arrow**), which showed necrosis. *SlSOD3* expression was somewhat higher in leaves 5 and 6 in H_72E. The values with the double asterisk (**; *p* < 0.01) and single asterisk (*; *p* < 0.05) were statistically significant at 1% and 5% levels compared to healthy-EC.

Three plants each from lines 72E and EC were infected with PSTVd. Leaf discs were harvested from leaves 5 and 6 (numbers from the bottom) at 4 wpi for RNA extraction, when symptoms of yellowing accompanied by vein necrosis started to develop in the leaves 5 and 6 of line 72E (Figure 8a). For comparison, the upper (leaves 7 and 8) and the lower (leaf 4) leaves showing no vein necrosis were also harvested and treated similarly. For the control, RNA samples were obtained in a similar way from three plants each of healthy 72E and EC lines. The assay was repeated twice, with each analysis consisting of three biological replicates collected from three plants per treatment.

Extremely high levels of miR398a-3p were specifically detected in leaf 6 of the PSTVd-72E plants showing vein necrosis (Figure 8b, arrow). In contrast, the mRNAs expression levels of *SlSOD2*, *S1SOD3*, and *S1SOD4* in the same leaves were significantly lower compared with those in the upper and the lower leaves in PSTVd-72E plants, as they were in the healthy-EC, PSTVd-EC, and healthy-72E plants (Figure 8c, arrow). A clear negative correlation was therefore present between the abnormally higher levels of miR398a-3p and significantly lower levels of *SlSOD3* mRNA in leaf 6 showing vein necrosis in line PSTVd-72E. This suggests that negative regulation of *SlSOD3* at the post-transcriptional level did occur in the leaves, as a result of the high levels of miR398a-3p. It is interesting to note that, as expected from Northern-blot analysis, elevated levels of miR398a-3p were observed in PSTVd-EC plants in leaves 4– 6, and that the expression levels of some *SlSOD*s, mRNAs (e.g., *SlSOD1* in leaf 6, *SlSOD3* in leaves 4–6, *SlSOD4* in leaf 6) were down-regulated to some extent (Figure 8b,c), however, these differences were much less pronounced compared with those in PSTVd-72E.

The levels of *SlSOD1*, *SlSOD2*, and *SlSOD4* mRNAs were also significantly decreased in leaf 6 of PSTVd-72E (Figure 8c, arrow) which showed necrosis. We have not yet fully explored this finding, but it is highly probable that the down-regulation of these *SlSOD*s was caused by the up-regulation of miR398, the negative regulator of these genes, because the up-regulation of miR398 in PSTVd-72E was already supported by the findings of our deep sequencing analysis of small RNAs (Table 1), and our preliminary RT-qPCR analysis indicated a high level of miRNA398 in leaf 6 of PSTVd-72E. It is necessary to further analyze the expression levels of miR398 in these leaves to clarify whether the high level of miR398 expression and down-regulation of *SlSOD1*, *SlSOD2*, and *SlSOD4* mRNAs are also involved in the onset of necrosis symptoms observed in line PSTVd-72E. It should be noted here that, although we have not yet identified the mechanism, the expression of *SlSOD*s, especially *SlSOD3*, was somewhat higher in leaves 5 and 6 in 72E, even without PSTVd infection.

### 3.9. ROS Production and Scavenging Activity in PSTVd-Infected Line 72E

**Figure 9:**
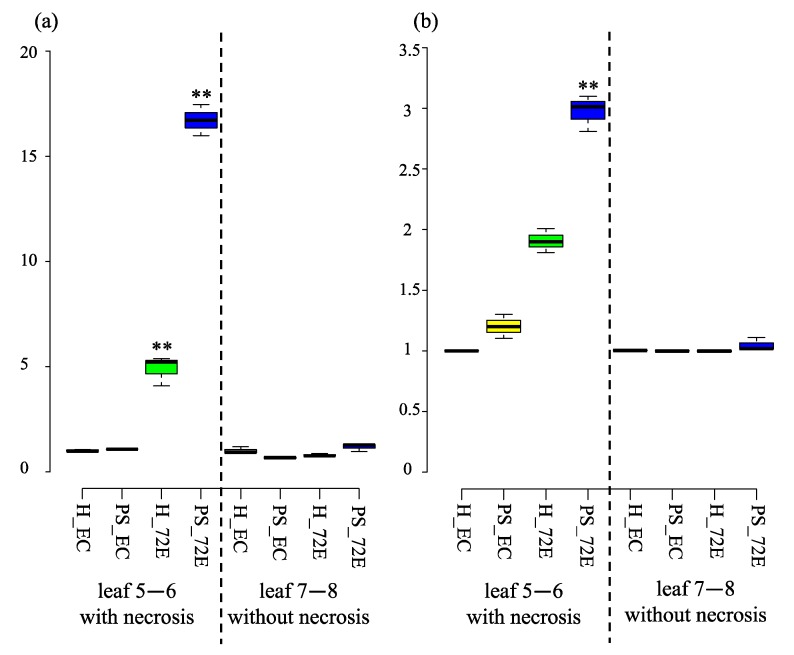
Relative ROS production (**a**) and relative ROS scavenging activity (**b**) in the leaves 5 and 6 (with necrosis) and leaves 7 and 8 (without necrosis). An unusually high level of ROS production and scavenging activity was found in leaves 5 and 6, showing necrosis in PSTVd-72E (PS_72E) compared with healthy-EC (H_EC) plants. A relatively high level of ROS production and scavenging activity was also detected in the leaves 5 and 6 of healthy-72E (H_72E) plants. Leaves of the same plants as described above were used for the analysis. The assay was repeated twice, and each analysis consisted of three biological replicates collected randomly from five plants per treatment. The center lines show the medians; the box limits indicate the 25thand 75thpercentiles as determined using R software; the whiskers extend 1.5-times the interquartile range from the 25thand 75thpercentiles; the dots represent outliers. The values with the double asterisk (**; *p* < 0.01) was statistically significant at 1% level compared to healthy-EC.

Using the same plants as described above, the activity of the ROS hydrogen peroxide was assayed in the healthy and PSTVd-infected 72E and EC lines during the period of 4–10 wpi. This is because PSTVd-72E plants had stopped growing and severe necrosis symptoms were underdeveloped. Leaves 5 and 6, which showed vein necrosis, and a mixture of upper leaves without necrosis (leaves 7 and 8) were selected for the analysis, because, as described above, PSTVd-72E plants started to show yellowing and vein necrosis in the leaves 5 and 6 at 4 wpi, but showed only leaf curling in leaf 7 and above. The highest activity was observed in leaves showing necrosis in PSTVd-72E plants. Interestingly, the activity was also high in the corresponding leaves of healthy-72E plants. Average hydrogen peroxide activity in the leaves showing necrosis in PSTVd-72E plants was ca. 15- and five-times higher than in healthy-72E and in healthy-EC or PSTVd-EC. A statistically significant difference (*p* < 0.01) was found between healthy-EC and healthy- and PSTVd-72E plants (Figure 9a; Table S2). In contrast, the activity was equally low in all samples of upper leaves without necrosis, indicating that lower leaves with necrosis of PSTVd-72E plants showed higher levels of hydrogen peroxide activity.

Hydrogen peroxide scavenging activity was also assayed in the same leaves in both healthy and PSTVd-72E and –EC plants. Average hydrogen peroxide scavenging activity was 20% in healthy-EC, 27.5% in PSTVd-EC, 33.6% in healthy-72E, and 44% in PSTVd-72E plants. A statistically significant difference (*p* < 0.01) was found between healthy-EC and PSTVd-72E plants (Figure 9b, Table S2), indicating that the leaves showing vein-necrosis in PSTVd-72E showed higher levels of hydrogen peroxide scavenging activity.

## 4. Discussion

In this experiment, by using RNAi-mediated matter, we produced a T3-generation of DCL2&4-knockdown ‘Moneymaker’ tomato lines, transformed with an IR construct consisting of partial sequences of tomato homologs *SlDCL2a*, *SlDCL2b*, and *DCL4*. One of these lines, named line 72E, was shown to contain a single copy of the transgene and to express high levels of transgene transcript and related siRNAs.

Line 72E, when challenged by PSTVd inoculation, started to show apical leaf curl at approximately 2 wpi and exhibited systemic leaf chlorosis, accompanied by vein-necrosis at 3–4 wpi. The plants subsequently stopped growing, developed more severe leaf necrosis (from lower to systemic), and finally died 4–5 months after inoculation, suffering from lethal systemic necrosis. This was in contrast to line EC, which was used as a control. Since the ‘Moneymaker’ tomato is a tolerant cultivar to PSTVd infection [10,72], line EC showed very mild leaf curl and stunting during the observation period. Therefore, RNAi-mediated down-regulation of *DCL2a* and *DCL4* expression changed the ‘Moneymaker’ tomato into being highly susceptible to PSTVd infection. Here we have to emphasize that in this experiment partial sequences of *DCL2a* and *2b* were used to construct a transgene, and the sequence was so similar to the other *DCL2*s (i.e., 2b, 2c, and 2d) that they could be also down-regulated in some extent at the post-transcription level in the line 72E. Actually, as shown in Figure S5, the expression level of *DCL2d* decreased to ~60% of the control, therefore, the word “DCL2” in the following sentences includes the other DCL2s either.

In line with the severity of disease symptoms, PSTVd level was significantly higher (1.5–3.0 times) in line 72E than in the other lines in early infection (until 2 and 3 wpi) (Figure 3), indicating that DCL2a and DCL4 play an important role in protecting tomato plants from PSTVd-induced symptom expression by suppressing the initial replication and accumulation of PSTVd.

By the analysis of PSTVd-sRNA using RNA-gel blot assay, lines EC, 51-3 and 82a were found to have accumulated two major bands of the sizes 21 and 22 nt and a weak band of 24 nt, whereas line 72E accumulated a dense 24 nt band and faint 21 nt band. The result was more evident in the data after deep sequencing: The number of PSTVd-sRNA reads of the sizes 21 and 22 nt species in line 72E decreased to ~66.7% and ~5% of those in line EC, respectively, and the 24 nt species in line 72E increased by ~1100% that in line EC.

Considering the commonly accepted concept that DCL1, DCL2, DCL3, and DCL4 generate 21 nt, 22 nt, 24 nt, and 21 nt sRNAs, respectively, the result clearly indicated that RNAi-mediated down-regulation of DCL2s and DCL4 resulted in a decrease of 21 and 22 nt species; 24 nt species, in contrast, increased significantly by relative superiority of DCL3 activity. The result is in line with the previous findings on the changes in the size distribution of virus- and viroid-derived sRNAs accumulated in *dcl2* and *dcl4* mutants and/or the knockdown lines [20,21,52,60,62]. The underlying mechanism behind this is a hierarchical interaction existing in the functions of DCLs in antiviral defense; i.e., DCL4 preferentially plays a major role in general. However, DCL2 is known to compensate for DCL4 function once DCL4 is destroyed or interfered [52,60,62]. Similarly, Katsarou et al. [21] reported, by using PSTVd-infected *dcl*(s) mutant lines of *Nicotiana benthamiana,* that the combined activity of DCL2 and DCL3 is important for anti-viroid defense. They presented a model that shows that DCL4 normally plays a major role in anti-viroid defense and suppresses the functions of DCL2 and DCL3. This was also the case in our experiment on the PSTVd-infected tomato hpDCL2/4i-72E line, in which the activity of DCL3 was significantly enhanced when expression of DCL2 and DCL4 was artificially suppressed.

Another dramatic increase found in 17 nt species in the 72E line was particularly interesting because a majority of them were originated from the upper strand of the pathogenicity region. The biogenesis is unknown, but a similar mechanism, reported by Zhu et al. [73], may be included, in which bidirectional processing of pri-miRNAs with branched terminal loops by *Arabidopsis spp.* DCL1 results in the production of 16–17 nt species, because viroids can form branched terminal loop structures and DCL1 was active in the plants.

As described above, RNAi-mediated knockdown of *SlDCL2*s and *SlDCL4* created a significant change in the size distribution of PSTVd-sRNAs. Nevertheless, the rate of decrease in 21 nt species (approximately 33%) was apparently smaller than that in 22 nt species (approximately 95%). This big difference seems to contradict the data obtained by RT-qPCR on the expression of all four *SlDCL*s; namely, the rate of decrease in *SlDCL2*s and *SlDCL4* expression in 72E line was approximately 50–60% of the empty cassette, not so different from each other. Taking the result that the rate of decrease in the transcription level of *SlDCL4* (producing 21 nt species) was almost the same as that of *SlDCL2a* (produces 22 nt species), and also the fact that DCL1 is also responsible for producing 21 nt species, the results may indicate that DCL1 is actually involved in the processing of viroid RNA and contributes to the production of 21 nt species of vd-sRNA, as suggested previously [74,75]. From this viewpoint, it is interesting to note that the expression level of *SlDCL1* in line 72E significantly up-regulated after PSTVd infection (Figure 6a). Alternatively, another possible explanation will be a significantly elevated level of expression found in *SlDCL4* after PSTVd infection (Figure 6d).

RT-qPCR analysis clearly indicated that PSTVd infection activated transcription level of *SlDCL4* in line 72E. On the other hand, the activation of transcription by PSTVd infection was observed not only in *SlDCL4* but also the other *SlDCLs* including *SlDCL2*s, indicating that expression of all the *SlDCL*s was activated by PSTVd infection. Since it was reported that the levels of *DCL1*, *DCL2*, and especially *DCL4* transcripts were increased significantly in a wild type tomato (cv. ‘Rutgers’) by the infection of citrus exocortis viroid (CEVd), another member of the genus *Pospiviroid* [76], it may be a general phenomenon for viroids, at least those in the genus *Pospiviroid*.

An important point here is that the sensitivity to PSTVd infection largely changed in line 72E, even though all the *DCLs* expression levels seemed to increase or somewhat recover after PSTVd infection. The result seems to create a question that cannot be explained by the expression levels of individual *DCLs*. An appropriate balanced expression of all the *DCLs* may be important to express an optimum anti-viroid defense response. Further analysis on the activities of DCL enzymes, for example, is necessary to clarify this point.

Changes in miRNA reads in the sRNA deep sequencing data were analyzed in silico, and several miRNAs were found to have been up- or down-regulated in PSTVd-72E compared with PSTVd-EC. Among them, the increased numbers of miR398 (770%) and miR398a-3p (868%) in PSTVd-72E were extremely interesting. MiR398 and miR398a-3p are stress-responsive miRNAs, known to be expressed in response to various stresses, that inhibit the expression of cytosolic and chloroplast-localized SODs [77,78] and copper chaperons for SOD (i.e., CCS1) [63], which scavenge harmful ROS. Northern-blot hybridization analysis clearly showed that miR398a-3p was detected exclusively from PSTVd-infected plants (both 72E and EC), with the intensity of the bands approximately five-times higher in PSTVd-72E compared with PSTVd-EC. These results, along with the observation that PSTVd-72E experienced severe systemic necrosis, strongly suggested that ROS are generated not only in PSTVd-72E but also in PSTVd-EC. The highest ROS production and scavenging activities were found in leaves showing necrosis in line PSTVd-72E, indicating that ROS were actively generated and then rapidly scavenging desperately in the plants. In contrast, even after PSTVd infection, the line EC did not show any visible necrotic symptoms or excessive ROS production. Therefore, the unusually higher levels of ROS production in PSTVd-72E seems to be a major reason for the development of severe necrotic reactions in these plants. More specifically, miR398 and 398a-3p must be down-regulated under normal conditions in order to enhance the function of SODs when ROS are produced following a viroid infection. However, as shown in Figure 8, the expression of miR398a-3p was particularly high in the leaves showing necrosis in PSTVd-72E (Figure 8b, leaf 6), and in response the expression of chloroplast-localized Cu/Zn-SOD2 mRNA (i.e., *SlSOD3* in tomatoes; Figure 8c, leaf 6 in *SlSOD3*), which is negatively regulated by miR398a-3p, significantly decreased. This indicates that miR398a-3p, which must, in theory, be down-regulated, is conversely elevated in leaves showing necrosis in PSTVd-72E. Misregulation of miR398a-3p in PSTVd-72E causes dysfunction of SlSOD3 localized in chloroplasts, resulting in the inability to regulate ROS scavenging, and subsequently leading to the development of severe systemic necrosis.

In conclusion, the results presented here clearly indicate that the tomato homologs of DCL2s and DCL4 provide strong but incomplete anti-viroid defenses and suppress viroid accumulation during the early stages of an infection. The replication of a highly-structured dsRNA-like hairpin RNA from a viroid genome serves as a PAMP and activates RNA-silencing targeting viroids. As a result, PSTVd-tolerant tomato cultivars, such as ‘Moneymaker’, continue to grow almost normally and show very few symptoms of disease. In contrast, the hpDCL2/4i-Moneymaker tomato line failed to defend initial viroid infection via RNA silencing, and as a result, allowed more aggressive replication and/or accumulation of viroids, which seemed to trigger excessive ROS production. Even in the PSTVd-tolerant tomato cultivar, our results indicated that, once the normal expression or function of DCL2s and DCL4 was destroyed, an unusually high state of expression emerged in stress-responsive miR398 and miR398a-3p, even after plants were infected with PSTVd and excessive amounts of ROS were generated. This abnormal situation, of course, leads to the inherently undesirable suppression of SOD mRNAs at the post-transcriptional level, impairing ROS-scavenging activity, and resulting in the development of severe systemic necrosis ultimately leading to plant death.

The production of ROS is an important defense reaction against pathogens that is rapidly induced upon recognition of a pathogen attack. Recent data from comprehensive and global transcriptome and metabolome analyses suggested that viroid infections trigger plant immune responses and result in the activation of various signaling pathways and associated activities, such as MAPK3, PR1, 1,3-beta-glucanase, and ROS biogenesis [10,72,79,80]. On the other hand, ROS are harmful substances that can cause significant damage to cell structures. Therefore, the proper management of ROS production and scavenging is very important when plants are protecting themselves against pathogen attacks. The results presented in this study identified several key factors (i.e., miR398, miR398a-3p, Cu/Zn-SOD1 and 2, CCS1, and ROS) in tomato plants which are responsible for causing necrosis, one of the most typical and serious disease symptoms induced by viroid infections, and revealed the existence of an unresolved relationship between DCLs, a key factor in RNA silencing against viroids, and the cell mechanism used to control ROS biogenesis. PSTVd-sensitive tomato cultivars, such as ‘Rutgers’, often develop various degrees of leaf and/or vein-necrosis, especially following infection by severe and lethal strains of PSTVd, CEVd, or tomato apical stunt viroid [81,82]. The observations presented here probably represent a general phenomenon that occurs in viroid–host interactions. Further elucidation of the molecular mechanisms underlying the development of viroid-induced necrosis will provide useful information with which it is possible to protect plants from severe damage resulting from viroid infections.

## Figures and Tables

**Table 1:** Changes in tomato sRNA levels in lines potato spindle tuber viroid (PSTVd) -72E and -EC.

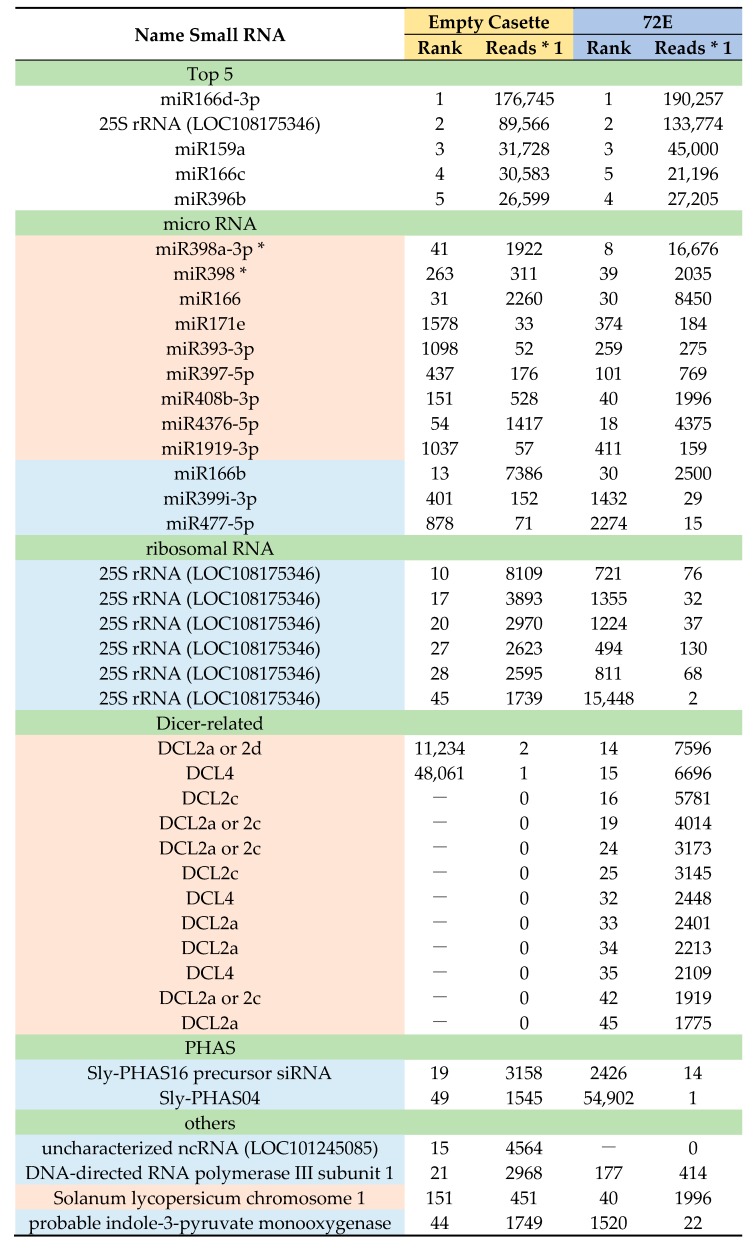

* This table shows a list of tomato sRNAs with more than 50 reads per million which changed more than three-times between PSTVd-EC and PSTVd-72E. Those up-regulated in PSTVd-72E were shown in **pink**, while down-regulated ones were in **blue**. MiR398 and miR398a-3p were up-regulated 770–868% in the 21 nt sRNA population from PSTVd-72E compared to those from PSTVd-EC. MiR398 and miR398a-3p have been reported to target Cu/Zn-SODs (CSD1, CSD2, and CCS1) in *Arabidopsis* spp. and possibly in the tomato. Levels of miR166b in line PSTVd-72E were only half of those found in PSTVd-EC.

## Supplementary Materials

The following are available online at https://www.mdpi.com/1999-4915/11/4/344/s1, Figure S1: Detection of transgene, transgene transcript, and transgene-derived siRNAs from three transgenic lines (51-6, 72E, and 82a) and control line (EC), Figure S2: Primary symptoms associated with potato spindole tubre viroid (PSTVd) infection in the DCL2/4i-72E and 82a Moneymaker tomato line, Figure S3: Northern-blot hybridization analysis to monitor the levels of potato spindle tuber viroid (PSTVd) accumulation in DCL2/4i-51-6, 72E, and 82a lines, Figure S4: RT-qPCR analysis of endogenous *SlDCL2b*, *2c*, and *2d* mRNAs in line 72E and EC before or after infection of potato spindle tuber viroid (PSTVd), Figure S5: Histograms of the number of potato spindle tuber viriod (PSTVd)-sRNA reads in 72E and EC (Emp in the figure), Figure S6: A comparison of hotspot pattern, Figure S7: In silico analysis of tomato miR398 and miR398a-3p target genes by psRNATarget software, Table S1: Names and sequences of PCR primers, Table S2: Relative ROS production and relative ROS scavenging activity of the 5th and 7–8th true leaves.
