# Supplementary material for: RNAi-Mediated Down-Regulation of Dicer-Like 2 and 4 Changes the Response of ‘Moneymaker’ Tomato to Potato Spindle Tuber Viroid Infection from Tolerance to Lethal Systemic Necrosis, Accompanied by Up-Regulation of miR398, 398a-3p and Production of Excessive Amount of Reactive Oxygen Species
Source: Viruses. 2019 Apr 13;11(4):344. doi: 10.3390/v11040344 (PMC6521110; doi:10.3390/v11040344)
Supplement: Supplementary file 1 [file viruses-11-00344-s001.zip › suplementary, Suzuki et al..docx]

**Supplementaly Tables**

**Table S1.** Names and sequences of PCR primers

**Table S2.** Relative ROS production and relative ROS scavenging activity of the 5th and 7–8th true leaves.


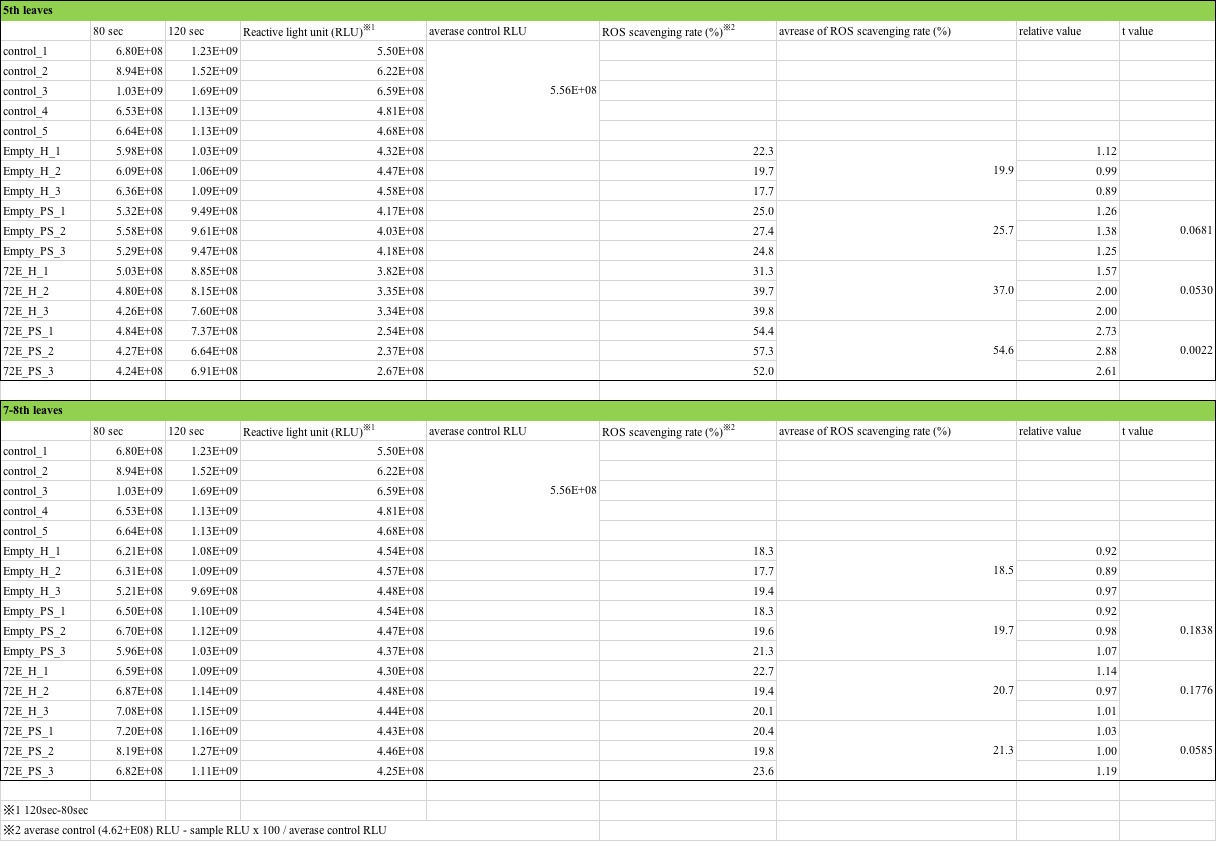


Reactive light unit (RLU) in this table means ROS production. ROS scavenging rate means ROS scavenging activity. AccuFLEX Lumi400 (Hitachi, Japan) was used for the mesurement.

**Sapplemetaly Figures**


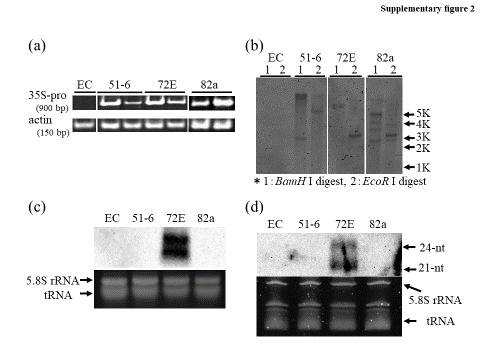


**Figure S1.** Detection of transgene, transgene transcript, and transgene-derived siRNAs from three transgenic lines (51-6, 72E, and 82a) and control line (EC). Panel (a) shows amplification of 35S-promoter sequence by PCR. Two each of individual plants were analyzed for the transgenic lines. Panel (b) shows detection of 35S-promoter sequence by Southern-blot hybridization. Genomic DNAs (~10 µg), extracted from three transgenic lines and the control line EC were separated in 1.0% agarose gel, transferred to nylon membrane and hybridized with a DIG-labeled cRNA probe for 35S-promoter. Lane 1 is *Bam*HI digest and lane 2 is *Eco*RI digest. The 72E line showed a single band either by *BamH*I or by *EcoR*I. Panel (c) and (d) show northern-blot hybridization analysis of transgene transcripts (c) and transgene-derived siRNAs (d) in the transgenic lines (hairpin DCL2/4-51-6, -72E, -82a) and control line EC. In panel (c), total RNAs (~10 µg), extracted from three transgenic lines and the control line EC were separated in 1.2% agarose gel, transferred to nylon membrane and northern hybridized with a DIG-DCL2/4 cRNA probe. The upper panel shows the hybridization signal and the lower panel shows the loading control stained with ethidium bromide. The 72E line showed a dense hybridization signal. In panel (d), total RNAs (~10 µg), extracted from three transgenic lines and the control line were separated in 8M-urea 12% polyacrylamide gel, transferred to nylon membrane and northern hybridized with a DIG-DCL2/4 cRNA probe. The upper panel shows the hybridization signal and the lower panel shows the loading control stained with ethidium bromide. The 72E line showed two dense hybridization signals of the size ~21- and 24-nt.


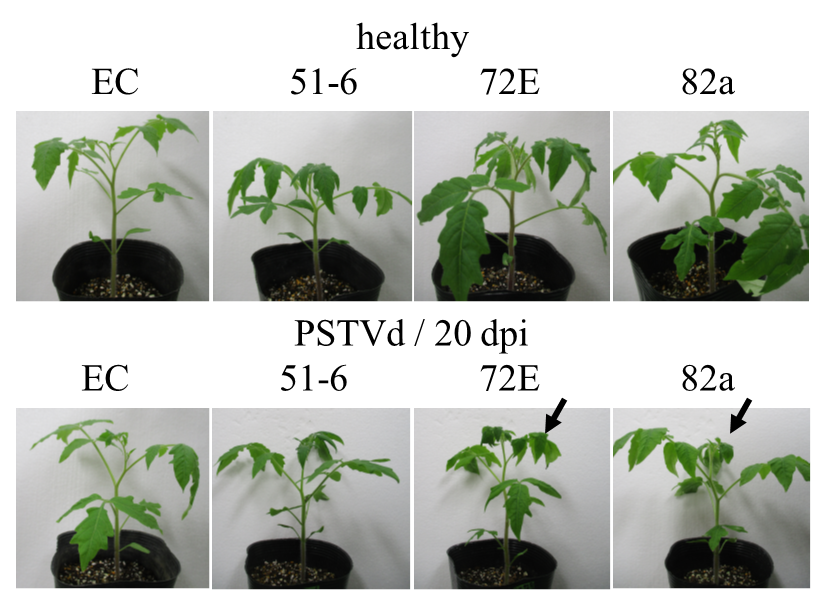


**Figure S2.** Primary symptoms associated with PSTVd infection in the DCL2/4i-72E and 82a Moneymaker tomato line. The arrows indicates mild leaf curling ~20 dpi.


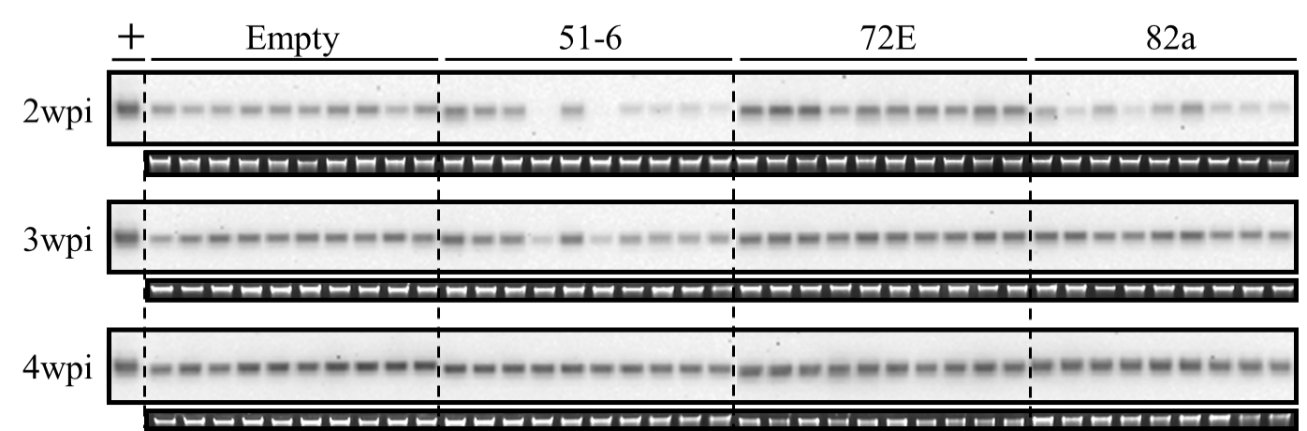


**Figure S3.** Northern-blot hybridization analysis to monitor the levels of PSTVd accumulation in DCL2/4i-51-6, 72E, and 82a lines. Total RNA extracted from the upper leaves of nine to ten individual tomato plants from each line at 2, 3, and 4 wpi was used to monitor the levels of PSTVd accumulation. PSTVd was detected in almost every inoculated plant, even at 2 wpi (10/10 plants infected in line EC, 8/10 in line 51-6, 10/10 in line 72E, and 9/9 in line 82a. One or two weeks later, all plants were infected.

**
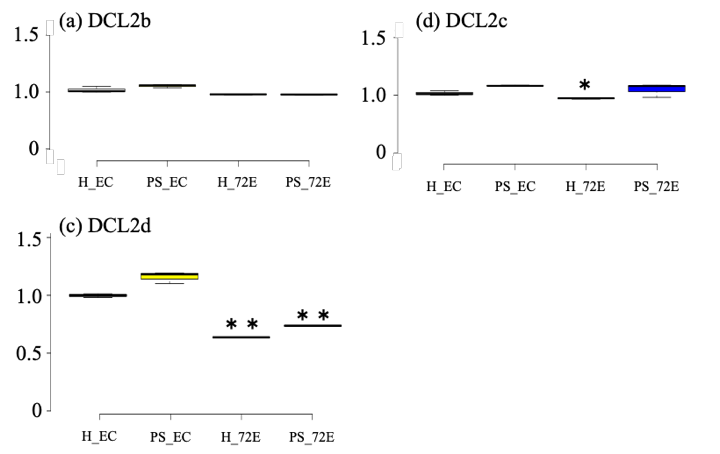
**

**Figure S4.** RT-qPCR analysis of endogenous *SlDCL2b*, *2c*, and *2d* mRNAs in line 72E and EC before or after infection of PSTVd.

Levels of *SlDCL2d* mRNA in line 72E before PSTVd-infection (H_72E) were significantly low comparing to those in line EC before PSTVd infection (H_EC), suggesting that endogenous *SlDCL2d* mRNA was also down-regulated in line 72E. The level was up-regulated a little after PSTVd-infection (PS_72E) but still low significantly. In contrast, levels of mRNAs of *SlDCL2b* and *2c* were significantly low even in line EC bofore or after PSTVd-infection, therefore, it was not apparent whether they are different in line 72E before or after PSTVd-infection. The value with double asterisk (**; *P* < 0.01) and single asterisk (*; *P* < 0.05) were statisctically significant at 1% and 5% level compared to healthy EC line. The value with “a” (a; *P* < 0.05) were statistically significant at 5% level compared to PSTVd EC line.


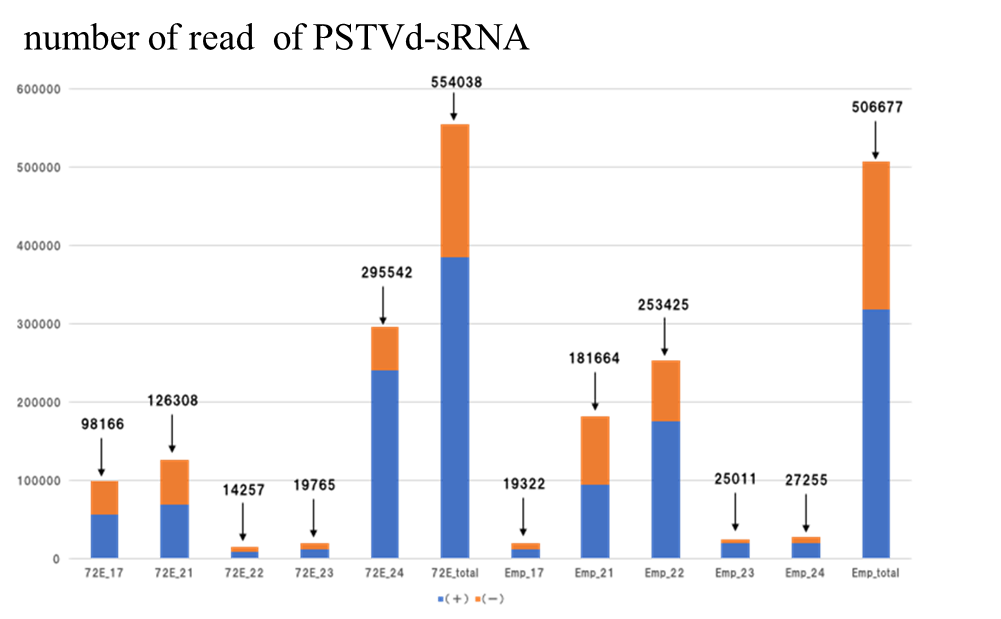


**Figure S5.** Histograms of the number of PSTVd-sRNA reads in 72E and EC (Emp in the figure). Five major PSTVd-sRNA size classes (i.e., 17, 21, 22, 23, and 24) were presented. The most abundant size class in line EC was 22-nt (50%), followed by 21-nt (36%), 24-nt (5%), and 23-nt (5%), however, the most abundant size class in line 72E, was 24-nt (53%), followed by 21-nt (23%), 17-nt (18%), 23-nt (4%), and 22-nt (3%).


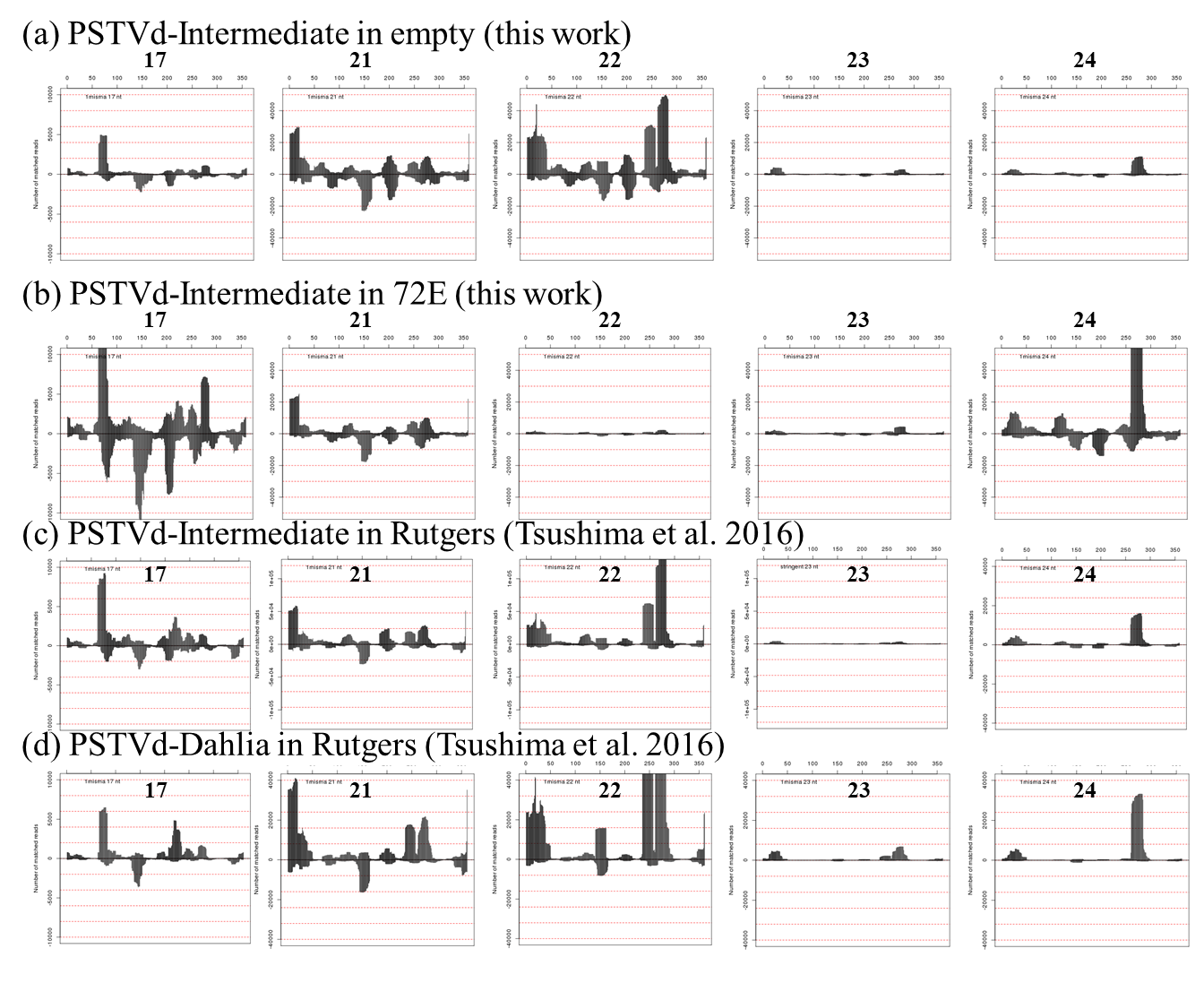


**Figure S6.** A comparison of hotspot pattern (size distribution and positive (+) / negative (–) ratio) of PSTVd-sRNAs of the size 17-nt, 21-nt, 22-nt, 23-nt, and 24-nt accumulated in the EC (a) and 72E (b) lines in this work, and in a similar analysis performed previously in Tsushima et al. [11]. Both of the analysis was performed by Hokkaido System Science Co., Ltd. (Sapporo, Japan) for small RNA sequence analysis (2Gb scale, paired end) using an Illumina HiSeq (Illumina, San Diego, CA, USA). Moneymaker tomato infected with PSTVd-intermediate was used in this work, and Rugers tomato infected with PSTVd-Intermediate and PSTVd-Dahlia was used in the previousl work. Nevertheless, the hotspot patterns seen in this work (a) was very similar to those in the earlier report (b and c), indicating a high degree of reproducibility among multiple deep sequencing analyses.


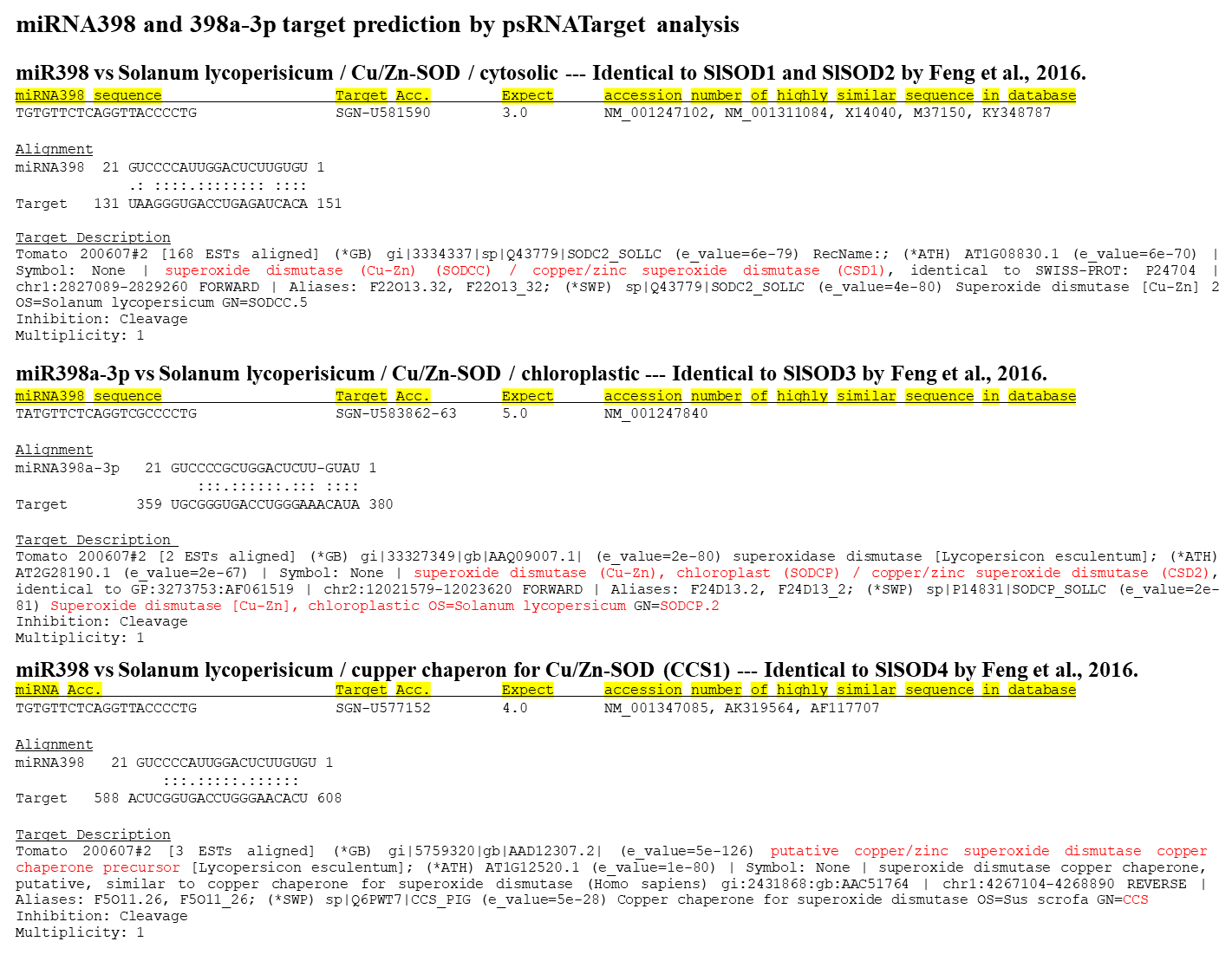


**Figure S7.** In silico analysis of tomato miR398 and miR398a-3p target genes by psRNATarget software.

Upper; The tomato miR398 sequence detected in this research was found to have a target sequence in tomato cytosolic Cu/Zn-SOD1 gene (SGN-U581590), which was the same to SlSOD1 and SlSOD2 by Feng et al. [66].

Middle; The tomato miR398a-3p sequence detected in this research was found to have a target sequence in tomato chloroplast-localized SOD2 gene (SGN-U583862-63), which was the same to SlSOD3 by Feng et al. [66].

Bottom; The tomato miR398 sequence detected in this research was also found to have a target sequence in tomato copper chaperon for SOD (CCS1) gene (SGN-U577152), which was the same to SlSOD4 by Feng et al. [66].
